# An efficient proteome-wide strategy for discovery and characterization of cellular nucleotide-protein interactions

**DOI:** 10.1371/journal.pone.0208273

**Published:** 2018-12-06

**Authors:** Yan Ting Lim, Nayana Prabhu, Lingyun Dai, Ka Diam Go, Dan Chen, Lekshmy Sreekumar, Louise Egeblad, Staffan Eriksson, Liyan Chen, Saranya Veerappan, Hsiang Ling Teo, Chris Soon Heng Tan, Johan Lengqvist, Andreas Larsson, Radoslaw M. Sobota, Pär Nordlund

**Affiliations:** 1 School of Biological Sciences, Nanyang Technological University, Singapore, Singapore; 2 Department of Anatomy, Physiology and Biochemistry, The Swedish University of Agricultural Sciences, Uppsala, Sweden; 3 Department of Oncology and Pathology, Karolinska Institutet, Stockholm, Sweden; 4 NTU Institute of Structural Biology, Nanyang Technological University, Singapore, Singapore; 5 Institute of Molecular and Cell Biology, Agency for Science, Technology and Research, Singapore, Singapore; Shantou University Medical College, CHINA

## Abstract

Metabolite-protein interactions define the output of metabolic pathways and regulate many cellular processes. Although diseases are often characterized by distortions in metabolic processes, efficient means to discover and study such interactions directly in cells have been lacking. A stringent implementation of proteome-wide Cellular Thermal Shift Assay (CETSA) was developed and applied to key cellular nucleotides, where previously experimentally confirmed protein-nucleotide interactions were well recaptured. Many predicted, but never experimentally confirmed, as well as novel protein-nucleotide interactions were discovered. Interactions included a range of different protein families where nucleotides serve as substrates, products, co-factors or regulators. In cells exposed to thymidine, a limiting precursor for DNA synthesis, both dose- and time-dependence of the intracellular binding events for sequentially generated thymidine metabolites were revealed. Interactions included known cancer targets in deoxyribonucleotide metabolism as well as novel interacting proteins. This stringent CETSA based strategy will be applicable for a wide range of metabolites and will therefore greatly facilitate the discovery and studies of interactions and specificities of the many metabolites in human cells that remain uncharacterized.

## Introduction

Low molecular weight metabolites play essential roles in most cellular processes, from the biogenesis of lipids, nucleic acids and proteins to cellular signaling and energy metabolism [1–3]. A key function of metabolites is their specific interaction with cognate proteins. Many such interactions are regulatory and act by modulating catalytic or binding activities of the target proteins. Metabolites also interact with enzymes as substrates and products. Due to their key roles in the cell, the dysregulation of metabolite-protein interactions is central to many different diseases [4–6]. Enzymes involved in the provision of limiting metabolites for cell proliferation are therefore frequently explored as drug targets in e.g. immunosuppressant or cancer therapy [7]. Traditionally, interactions between proteins and metabolites/second messengers were discovered using focused biochemical work, eventually depending on the isolation of the target protein in a purified form. Biophysical binding and activity studies, together with structural work on purified proteins, have yielded detailed insights into many such interactions [8, 9]. More recently, affinity proteomics that use modified low molecular weight ligands linked to affinity resins has in special cases enabled the identification of novel metabolite and second messenger interacting proteins in lysates [10–12]. Using activity-based protein profiling (ABPP), covalent probes applied in competitive experiments have allowed for the discovery of lipid and cholesterol-interacting proteins in intact cells [13, 14]. However, for many metabolites, it is challenging to synthesize relevant chemical probes and the addition of linkers or reactive moieties to metabolites may alter their biochemical characteristics, thus modulating their ability to bind to the anticipated targets. Very recently, limited proteolysis-coupled mass spectrometry (LiP-MS) was applied to systematically identify metabolite-protein interactions in *E*. *coli* lysates [15]. This method has the potential to also identify/confirm binding regions of metabolites but when very large numbers of non-expected metabolite binding sites were discovered in this study, additional filtering might be needed to provide the most relevant set of interactions. Although there has been significant progress in the development of methods to study protein-metabolite interactions at the proteome level, the interactions of relatively few metabolites have been successfully studied, suggesting that significant technical challenges remain. For the future, improved understanding of the stringency of current methods and the development of label-free methods for in-cell studies that yield minimal bias to the cellular machinery, are of particular interest.

**Fig 1:**
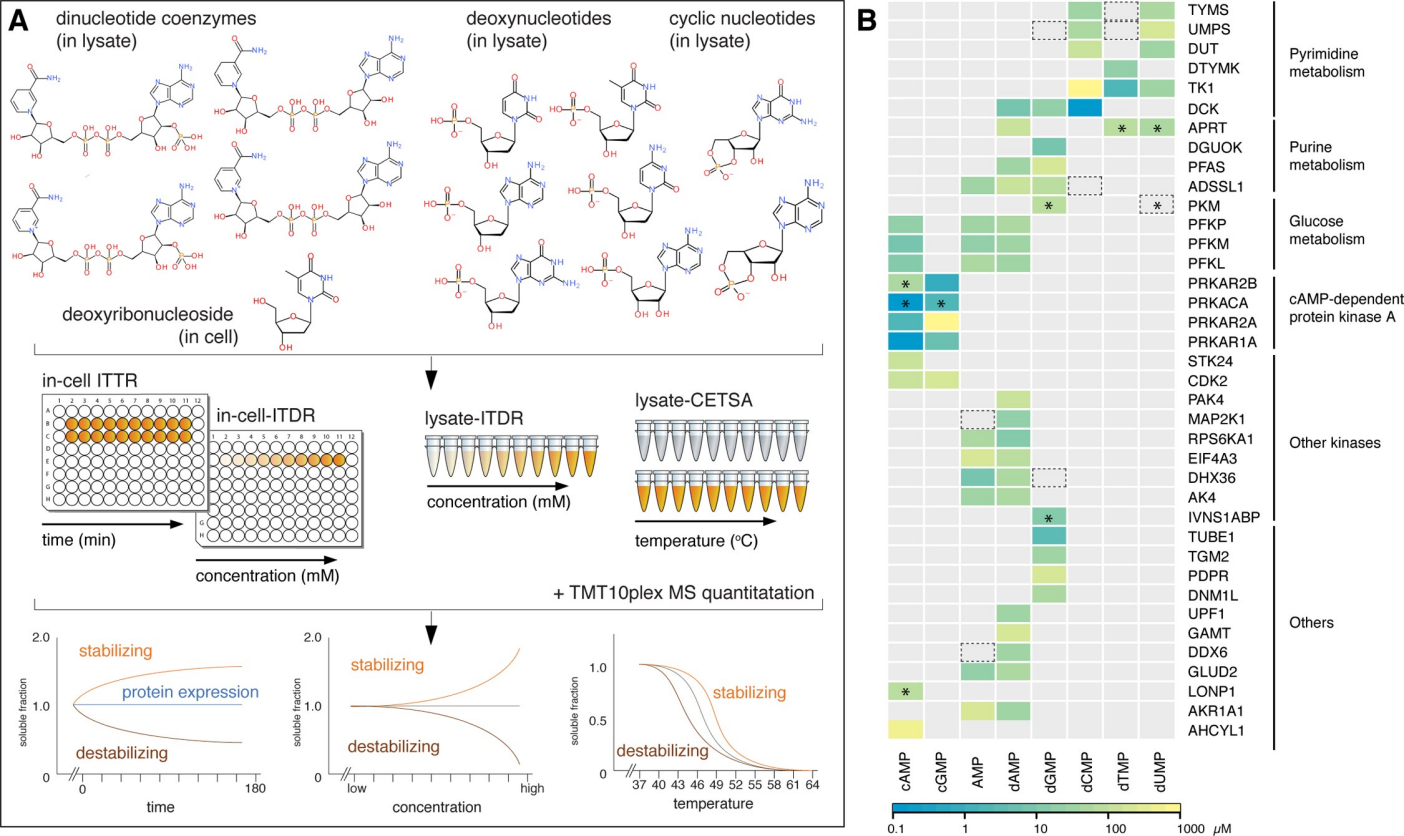
Optimized CETSA workflow for profiling protein-nucleotide interactions in whole cell and lysate conditions, and relative affinities and specificities of the eight nucleotides for their identified hit proteins in lysate-ITDR experiments. (A) ITDR and melt curves workflows were applied to lysate experiments and in-cell ITDR and ITTR to whole cell experiments as a proxy for tracking the uptake and conversion of metabolite precursors *in vitro*. (B) Colour scale represents the minimal dose threshold (MDT) (μM) of the isothermal dose response at 52°C, with the lowest MDT (therefore higher relative affinity) in blue and the highest MDT (therefore lower relative affinity) in yellow. Hit proteins are grouped according to their associated biochemical pathways and function (black lines). Asterisks (*) indicate destabilizing hits. Black dotted boxes indicate proteins with similar ITDR responses that did not meet the hit selection criteria.

In the present work we use a stringent strategy for the systematic discovery and characterization of interactions of nucleotide metabolites with the human proteome. We explore our recently developed cellular thermal shift assay (CETSA) to study interactions of human proteins with key nucleotide-based metabolites (Fig 1A) [16, 17]. CETSA is a biophysical technique based on the discovery that heat-induced protein unfolding leads to rapid precipitation in the cellular environment. The amount of folded protein after the heating step is estimated by quantifying the specific protein in the soluble fraction. This allows for the generation of protein melting curves and thereby ligand-induced thermal shifts for specific proteins in lysates and cells. CETSA has previously been shown to give stringent data for higher affinity drug interactions [18]. Although there are some protein-metabolite interactions that are strong including some having covalent contributions, most such interactions are relatively weaker than protein-drug interactions and are in the higher nanomolar or micromolar range. Although ATP interactions in lysates have previously been profiled with an early less stringent implementation of CETSA [19], this data was not used to assess specificities and confirm false positive rates. The general usefulness of CETSA to discover and resolve specificities of metabolite interactions at the proteome level therefore remains unknown. We have developed an improved strategy for proteome-wide mass spectrometry implementation of CETSA (MS-CETSA) to rapidly map protein-metabolite interactions in lysates and cells. The improved CETSA implementation confirmed known direct interactions, validated many predicted but experimentally uncharacterized interactions, and discovered novel cognate interactions with the nine different nucleotides. Subsequently we evaluated the dose and time-dependence of CETSA signals in thymidine-exposed cells, revealing the internalization of thymidine and the ensuing interactions of a cascade of thymidine metabolites with enzymes in the deoxyribonucleotide synthesis pathway, as well as several novel interaction partners for these metabolites.

## Materials and methods

### Reagents and cell culture

Reagents were purchased from Sigma-Aldrich, and cell culture media from Invitrogen unless otherwise stated. Phosphate buffered saline (PBS) and EDTA-free protease inhibitor cocktail was purchased from Nacalai Tesque. K562 cells were sourced from ATCC and cultured in RPMI medium supplemented with 10% fetal bovine serum (FBS, Biowest), 100 units/mL penicillin and streptomycin, non-essential amino acids, and 0.3 g/L L-glutamine. The cells were expanded to a maximum of 1 x 10^6^ cells/mL.

### Lysate-CETSA melting curves/ITDR_CETSA_ for MS

The cells were harvested, washed in PBS and re-suspended in 1x kinase buffer (50 mM HEPES, pH 7.5, 5 mM beta-glycerophosphate, 0.1 mM sodium orthovanadate (Na_3_VO_4_), 10 mM MgCl_2_, 2 mM TCEP, 1X protease inhibitor cocktail). The suspension was subjected to two freeze-thaw cycles with liquid nitrogen, followed by mechanical shearing with the syringe and a final freeze-thaw cycle. The cell debris was removed by centrifugation at 20,000 g for 20 min at 4°C. For the lysate-CETSA experiment, the lysate were spiked with metabolites to the final concentration of 1 mM or with same volume of PBS (control) and incubated at room temperature for 3 min. The spiked lysate was divided into 10 aliquots for heat treatment at the respective temperatures for 3 min in a 96-well thermocycler (Applied Biosystems), followed by 3 min at 4°C. The lysate was centrifuged at 20,000 g for 20 min at 4°C and the supernatant was transferred into microtubes for downstream analysis such as western blot or mass spectrometry.

For the lysate-ITDR_CETSA_, the metabolites were serially diluted in PBS prior to addition to the lysate. The spiked lysate was divided into 10 aliquots for heat treatment at 52°C for 3 min in a 96-well thermocycler, followed by 3 min at 4°C. The lysate was centrifuged and supernatant was transferred for further analysis as aforementioned.

### In-cell-ITDR_CETSA_ and ITTR_CETSA_ for MS

For the in-cell dose-response experiment, the cells were re-suspended in complete media and treated with a 4-fold dilution series of thymidine, with 25 mM as the highest concentration. The treated cells were washed after 3 hrs exposure and heated at 52°C for 3 min in a 96-well thermocycler, followed by 3 min at 4°C. The cells were lysed post-heat treatment according to the aforementioned procedure. For the in-cell time-dependent experiment, thymidine was added to the cells at 10 mM for a range of duration up till 120 min. The treated cells were washed, heated at 37°C or 52°C for 3 min, followed by 3 min at 4°C.

### pH buffer-exchange lysate-CETSA

Lysates were buffer-exchanged with PD-10 desalting columns (GE Healthcare) and eluted with the aforementioned kinase buffer containing either 50mM MES pH 6.5, 50mM HEPES pH 7.5, or 50mM HEPES pH 8.5, seven fractions per pH condition. The 2 samples with the highest protein concentration were combined for the CETSA heat pulse.

### Western blot-CETSA

Cells or lysates were treated with compounds or metabolites prior to CETSA heat pulse. The soluble protein fractions were prepared as aforementioned, then denatured and boiled in reducing NuPAGE LDS sample buffer, resolved on NuPAGE gels and transferred with the iBlot system (Invitrogen) onto PVDF membrane. The membrane was blocked in 5% skim milk for 15 min and incubated with primary antibodies: anti-PFKM and anti-RIA (Origene); anti-RIIB (Thermo Scientific); anti-beta-actin (Santa Cruz); anti-ABCF2 (Atlas Antibodies) and anti-RRM1 (Sigma Aldrich) overnight at 4°C. The membrane was washed with 0.05% Tween20-PBS, and probed with the appropriate secondary antibody: anti-DDK conjugated to horseradish peroxidase (HRP) (Origene); anti-mouse IgG-HRP and anti-rabbit IgG-HRP (Thermo Scientific) at room temperature. The chemiluminescence signal was developed with Clarity ECL blotting substrate and imaged with ChemiDoc MP Imaging System (Bio-Rad).

### Protein digestion and processing for MS

The protein content in the supernatants after lysis was measured using BCA assay. The protein samples were subject to reduction and denaturation with 20 mM TCEP and 0.1% (w/v) RapiGest at 55°C for 20 min and then to alkylation with 55 mM CAA at RT for 30 min. Proteins were first digested with LysC for 3–4 hrs then digested with trypsin overnight at 37°C. After digestion, the RapiGest was hydrolyzed with 1% TFA at 37°C for 45 min. The samples were then centrifuged at 20,000 g for 10 min and the supernatants were collected and dried using a centrifugal vacuum evaporator. The samples were solubilized in 100 mM TEAB at a final concentration of 1 μg/μl. 25 μg of the digested peptides was then labeled with Tandem Mass Tags -10plexTMT (Pierce). The labeling was carried out in 100 mM TEAB buffer for at least 1 hr before being quenched with 1M Tris, pH 7.4. The labeled samples were subsequently desalted using C18 Sep-Pak cartridge (Waters) and were pre-fractionated into 96 fractions using a high pH reverse phase Zorbax 300 Extend C-18 4.6 mm x 250mm (Agilent) column and liquid chromatography ÄKTA Micro (GE) system.

### LC-MS analysis

Following high pH offline separation, the samples were dried using vacuum centrifuge and 20 pooled fractions from each experiment were subjected to mass spectrometry analysis using reverse phase liquid chromatography Dionex 3000 UHPLC system connected to a Q Exactive mass spectrometer (Thermo Scientific). Each fraction was separated on 50 cm x 0.75 mm Easy Spray column (Thermo Scientific) in an 80 min gradient with a pre-programmed mixing of solvent A (0.5% acetic acid in water) and solvent B (80% acetonitrile, 0.5% acetic acid in water). The following acquisition parameters were applied: Data Dependent Acquisition with survey scan of 70,000 and AGC target of 3e6; Top12 MS/MS 35,000 and AGC target of 1e5; Isolation window 1.6 m/z. Peak lists for subsequent searches were generated in Proteome Discoverer 2.0 software (Thermo Scientific) using Mascot 2.5.1 (Matrix Science) and Sequest HT (Thermo Scientific) together with concatenated forward/decoy Human-HHV4 Uniprot database (88559 entries). Search parameters: MS precursor mass tolerance 30 ppm, MS/MS 0.06 Da, maximal 3 missed cleavages; static modifications: Carbamidomethyl (C); variable modifications: Oxidation (M), Deamidated (NQ), Acetyl N-terminal protein. False discovery rate estimation with 2 levels: Strict = 1% FDR, Medium = 5% FDR.

### Protein quantification and CETSA data processing

Reporter ion intensities and peptide identification for each peptide-spectrum match were extracted from MS/MS scans in Proteome Discoverer 2.0. Only unique peptides were used for the abundance quantification of the associated proteins. Protein groups with quantification information were exported and fed into an in-house developed CETSA data processing package in R environment (https://github.com/nkdailingyun/mineCETSA) for data extraction, clean-up and normalization, curve fitting and plotting.

For CETSA melting curve experiments, the fold-changes of any given protein across the heating temperature range were quantified by using the 37°C temperature condition as the reference (i.e., a constant value of 1). In contrast to the selection of a “typical melting curves” subset for data normalization as reported by Savitski et al. [18], we included all the quantified proteins to calculate the global reference medians. To perform data normalization, a fitting factor vector was first derived from each dataset: The median fold-change values for all the quantified proteins at each of the ten temperature points were calculated. These median values were fitted into a sigmoidal shaped curve to represent the averaged melting trend of the whole proteome. Thus, a 10-element fitting factor vector was calculated by dividing the fitted median value over the original median value at each of the ten temperature points. Specifically, the curving fitting was implemented with the four-parameter log-logistic nonlinear regression model by using LL.4() function from the ‘drc’ package:

$$f(T)=c+\frac{d-c}{1+e^{b(\log(T)-\log(e))}}$$

Where *T* is the temperature, *f(T)* is the fold-change value at temperature point *T*, and *b*, *c*, *d*, *e* is constants representing slope, lower limit, upper limit, and Tm value, respectively.

Next, a scaling factor was introduced to correct for possible differences in baseline signals among the comparing experimental conditions after each independent fitting. It was defined as the factor that the fitted median value at 37°C temperature would be multiplied with, to regain a common constant value of 1. This scaling factor was multiplied to each of the element in the vector of fitting factors to generate a new 10-element vector of normalization factors.

Finally, the data normalization was achieved by applying the respective normalization factors in the vector to the protein fold-change values of each temperature points in the respective experimental conditions.

### ITDR_CETSA_/ITTR_CETSA_ experiment, dose/time-response curve fitting and analysis

For IsoThermal Dose Response-CETSA (ITDR_CETSA_) (and similarly IsoThermal Time Response-CETSA (ITTR_CETSA_)) experiments, the protein fold-changes across the different concentrations of treating compound were quantified by reference against the sample treated with the lowest compound concentration or buffer-only condition. Data global normalization was achieved by adjusting the median fold change to be 1.0.

The fold-change represents the protein stability change when treated with the corresponding concentrations of compound in ITDR_CETSA_ experiment or at the corresponding time points in ITTR_CETSA_ experiment. To better determine fold-change cutoff level for hits selection, we first went on to determine the baseline variance from each experiment, i.e., the experiment-wise random technical fluctuation level. The median absolute deviation (MAD) scheme applied on the lowest three concentration groups of all the quantified proteins was used to estimate the baseline variance. We used the value of median +/- 2.5*MAD as cutoff for baseline variance, which is the minimal responsive level required when protein potentially binding to the test compound.

For any proteins to be considered confidently stabilized or destabilized by the compound, we required the fold-change value at any of the highest three compound concentration points should have at least 30% change when compared to minimum responsive level. We further required that the sigmoidal curve fitting to have an *R*^2^ >0.8, and the protein abundances were quantified by at least three peptide spectrum matches (PSMs). As illustrated in S1A Fig, for any ITDR_CETSA_ hits, we could therefore estimate the minimum dose threshold (MDT), which is the lowest compound concentration required to confidently pass the baseline level.

### Euclidean distance-based scoring scheme for hit ranking and data visualization

For CETSA melting curve analysis involving duplicates of control vs treatment conditions (highly recommended to avoid false positive target identifications), typically more than 75% of all the quantified proteins could be reproducibly identified in all the four independent mass spectrometry runs (i.e., defined as the complete subset), and at least 10% in three out of four runs. Therefore, out of the cumulatively identified proteins, 90% of them, which correspond to more than 5000 proteins, constituted the main body of proteins for downstream analysis and curation. To quantify both protein thermal shift and inter-replicates reproducibility, a Euclidean distance score (EDS) was derived for each protein belonging to the complete subset, in the following manner: The pairwise Euclidean distance (ED) between any two melting curves in an Euclidean 10-space was first calculated using the following formula:

$$ED=\sqrt{\sum_{i=1}^{10}{\left(f_{1}(T_{i})-f_{2}(T_{i})\right)}^{2}}$$

Where *f*_*1*_*(T*_*i*_*)* and *f*_*2*_*(Ti)* represents the protein fold-change value at temperature point *T*_*i*_ for pairwise melting curve 1 and 2, respectively.

The sum of inter-treatment Euclidean distance (*ED*_*inter-treatment*_) indicates the extent of change (i.e. apparent shift) of compound-treated protein melting curve from vehicle-treated counterpart, while the sum of inter-replicate Euclidean distance (*ED*_*inter-replicate*_) indicates the variance (i.e., experimental reproducibility) of protein melting curves from the same treatment in replicate runs. Subsequently, EDS was calculated using the formula below:

$$EDS=\frac{\sum ED_{inter-treatment}}{10^{\sum ED_{inter-replicate}}}$$

By definition, a larger EDS value indicates a reproducible and significant thermal shift when compared to the control condition, i.e., potential protein hits of the compound.

The CETSA melting curves for individual proteins were subsequently fitted using LL.4() function and visualized by plotting in a combined PDF file. The plots were presented in descending EDS order to allow easier inspection of potential hits. Textual annotation of the associated unique peptide and PSM number for each protein from each mass spectrometry run could be provided and served as supporting information.

### Pathway analysis and functional annotation

The Uniprot IDs of the hit proteins were submitted to the Reactome database for pathway analysis [20]. Proteins that were not annotated in the Reactome were manually curated based on gene-ontology (GO) terms from the Uniprot Knowledgebase [21]. The known interactions of metabolites and proteins were retrieved from HMDB database, Uniprot Knowledgebase and primary literatures. The interactions were imported into Cytoscape (v.3.4.0) for visualization [22].

### Expression and purification of recombinant protein

The gene encoding human Thymidylate Synthase (TYMS) (NM_001071.2) and ABCF1 (BC034488.2) was subcloned into pNIC28-Bsa4 vectors and expressed in Rosetta BL21-DE3 Escherichia coli (Novagen) in Terrific Broth media supplemented with 50 μg/ml of kanamycin and 34 μg/ml chloramphenicol. Cells were grown at 37°C until OD_600_ nm reached about 2.0 and induced with 0.5 mM isopropyl-beta-D-1-thiogalactopyranoside (IPTG) at 18°C overnight. The cells were harvested by centrifugation and resuspended in lysis buffer (100 mM HEPES, 500 mM NaCl, 10 mM imidazole, 10% (v/v) glycerol and 1 mM TCEP at pH 8.0) supplemented with 1:1,000 (v/v) EDTA-free protease inhibitor cocktail (Calbiochem) and 125 U/ml of Benzonase (Merck). Cells were lysed by sonication on ice at 70% amplitude, 3 s on/off for 3 min. The lysate was clarified by centrifugation at 47,000 g for 25 min at 4°C, and the supernatant was filtered through a 1.3 μm syringe filter. The cell-free extract was loaded on a pre-equilibrated HisTrap^TM^ HP column (GE Healthcare) in IMAC wash buffer 1 (20 mM HEPES, 500 mM NaCl, 10 mM imidazole, 10% (v/v) glycerol and 1 mM TCEP at pH 7.5) and subsequently washed with 20 column volumes (CVs) of IMAC wash buffer 1 and 15 CVs of IMAC wash buffer 2 (20 mM HEPES, 500 mM NaCl, 25 mM imidazole, 10% (v/v) glycerol and 1 mM TCEP at pH 7.5). Bound protein was eluted with 5 CVs of elution buffer (20 mM HEPES, 500 mM NaCl, 500 mM imidazole, 10% (v/v) glycerol and 1 mM TCEP at pH 7.5) and loaded onto a HiLoad 16/60 Superdex-200 column (GE Healthcare) pre-equilibrated with buffer (20 mM HEPES, 300 mM NaCl, 10% (v/v) glycerol, and 1 mM TCEP at pH 7.5). Based on NuPAGE gel results pure protein fractions were pooled and concentrated using centrifugal driven filter concentrators (Sartorius Stedium Biotech). The protein concentration was determined by the absorbance at 280 nm using a Nanodrop spectrophotometer (Thermo Scientific).

### Thermal shift *in vitro* assay on recombinant protein

The assay was performed on the iCycler iQ Real Time PCR Detection System (Bio-Rad), using the 96-well thin-wall PCR plate (Bio-Rad). A total volume of 25 μl of sample containing 0.2 mg/ml protein, compounds and 5X Sypro-orange dye (Invitrogen) was dispensed into the 96-well plate. The plates were sealed with Microseal B adhesive sealer (Bio-Rad) and heated in iCycler from 25°C to 80°C (56 heating cycles, in 1°C/30sec increments per cycle). Fluorescent filter used for Sypro Orange measurements was *λ*_*excitation*_ = 492 nm and *λ*_*emission*_ = 610 nm. The calculation of the midpoint of the curves (*T*_*m*_) was performed using the software package XLfit from IDBS within Microsoft Excel.

### TYMS enzymatic assay

Enzymatic activity of recombinant human TYMS was measured spectrophotometrically at 340 nm by monitoring the absorbance change during the conversion of 5,10-methylenetetrahydrofolate to dihydrofolate using an Infinite M200 spectrometer (Tecan). Measurements were carried out at room temperature and in a buffer of 50 mM Tris at pH 7.5 and 150 mM NaCl. Initial velocities were measured with 250 nM of purified protein, 100 μM 5,10-methylenetetrahydrofolate and 100 μM dUMP in the presence of compounds. Initial rates and activity were analyzed with the software package Prism (GraphPad Software).

### Crystallization and structure determination

TYMS protein was crystallized in sitting drops comprising equal volume of protein (about 24 mg/ml) and reservoir solution at 20°C. The crystallization condition was composed of 0.1 M sodium cacodylate at pH 6.5 and 15% PEG 4000. Crystals were soaked with 5 mM dCMP in cryo-protectant buffer containing 0.1 M sodium cacodylate at pH 6.5 and 15% PEG 4000 and 10% DMSO for 15 min, followed by flash frozen in liquid nitrogen. Data collection was performed on beamline 13B1 at National Synchrotron Radiation Research Center in Taiwan. The structure was solved by molecular replacement using Phaser with the hTYMS-dUMP-raltitrexed structure (PDB code 1HVY) as the search model. Structure was refined with phenix.refine. Ligand structures and restraints files were generated using eLBOW. The data collection parameters and refinement statistics are summarized in S10 Table. The crystal structure was deposited with the RCSB Protein Data Bank under the accession code 5WRN.

### Alphascreen-CETSA for lysate TYMS in cell lysate

K562 cells were harvested and lysed with three freeze-thaw cycles and mechanical shearing and then re-suspended in kinase buffer to a concentration of about 10 million cells/ml. The metabolite (dCMP, dUMP or dTMP) was prepared in water and added into the wells of a 96-well PCR plate at different concentrations. 5 μl of the cell lysate was added to the PCR wells containing the metabolite and incubated for the indicated time points. The samples were heated at 52°C using a thermal cycler for 3 min and then cooled for 3 min at room temperature. The samples were mixed and diluted with 10 μl Alphascreen Surefire lysis buffer (2.5X) (PerkinElmer). The lysate was mixed thoroughly and 3 μl was transferred to a 384 proxiplate (PerkinElmer). A reagent mix was prepared in 5X immunoassay buffer (PerkinElmer) with the antibodies, donor and acceptor beads. The 1.5X reagent mix contained 0.6 nM of mouse monoclonal anti-TYMS IgG (Santa Cruz), 1.5 nM rabbit polyclonal anti-TYMS IgG (Proteintech), 60 μg/ml anti-mouse IgG alpha donor beads (PerkinElmer) and 15 μg/ml of anti-rabbit IgG AlphaLISA acceptor beads (PerkinElmer). 6 μl of reagent mix was added to the samples and the plate was incubated in the dark overnight. The alphascreen readings were taken using an Envision plate reader (PerkinElmer), curves were plotted using the Graphpad software and fitted using the sigmoidal dose-response (variable slope) equation.

### Glucose and lactate metabolism assays

Cells were incubated in 1% dialyzed FBS, 5 mM glucose RPMI with cAMP activator/analog: 0.5 mM forskolin, or 2 mM bucladesine (Db-cAMP) (Biolog), or 2 mM 8-bromoadenosine 3’5’-cyclic monophosphate (Br-cAMP) (Biolog), up to 24 hrs. The supernatant was harvested; thereafter the cells were washed twice in 1X PBS. The supernatant and lysate were prepared and assayed using the Glucose-Glo, Glucose-Uptake-Glo, Lactate-Glo kit as per assay instructions (Promega). Data is presented as mean and standard deviation of 2 biological replicates and compared using paired two-tailed Student’s t-test.

### Measurement of cAMP and NADPH levels in lysates/lysates from treated cells

Lysates were treated with 1mM cAMP or NADPH up to 24 hrs at room temperature. Lysates treated with 1mM cAMP were diluted 5x in cAMP assay buffer (R&D Systems) and cAMP levels were measured against a standard curve as per assay instructions. Cells treated with cAMP activator/analog were lysed in cAMP assay buffer at 4°C and the cAMP levels were measured similarly in the neat lysates. Lysates treated with NADPH were diluted 10x in lysis buffer and relative NADPH levels were measured using the NADP/NADPH Glo kit as per assay instructions (Promega).

## Results

### An efficient proteome-wide MS-CETSA isothermal dose response (ITDR_CETSA_)-based hit generation protocol reveals prevalence of stabilizing interactions of nucleotide metabolites with proteins

**Fig 2:**
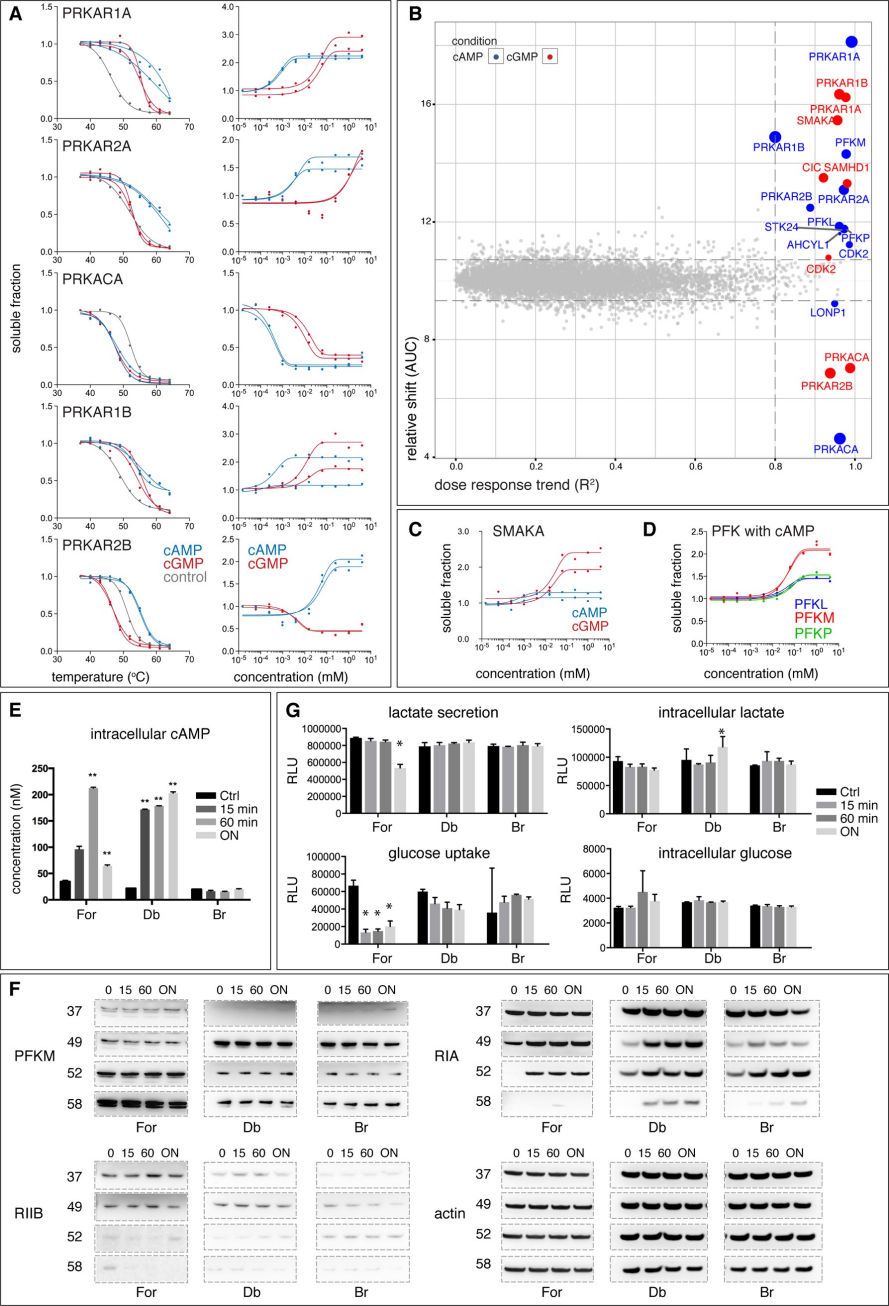
CETSA shifts and ITDR_CETSA_ with cAMP and cGMP in K562 lysates. (A) CETSA shifts (left) and ITDR_CETSA_ (right) for subunits of cAMP-dependent protein kinase A (PKA) (regulatory: PRKAR1A, PRKAR2A, PRKAR1B, PRKAR2B and catalytic PRKACA), with cAMP (blue), cGMP (red) and control untreated CETSA curves (grey) measured in K562 cell lysates. (B) ITDR_CETSA_ hit plot with AUC (area under the curve) versus R^2^ plot of ITDR_CETSA_ curves from cAMP/cGMP-treated conditions. Each dot represents the average AUC (relative thermal shift) and R^2^ ITDR_CETSA_ (dose response trend) measurements per protein from two replicates. The size of the dot negatively correlates with the MDT. (C) ITDR_CETSA_ for small membrane A-kinase anchor protein (SMAKA) with cAMP and cGMP. Colors as in Fig 2A. (D) ITDR_CETSA_ for homologs of phosphofructokinase (PFK)—PFKM (muscle), PFKL (liver), PFKP (platelet) with cAMP. Data is presented as two individual technical replicates for each protein. Cells were stimulated for the indicated durations (0, 15, 60 mins and overnight (ON)) with 0.5 mM of forskolin (For), 2 mM of Db-cAMP (Db) or Br-cAMP (Br), the samples were used to measure (E) cAMP level in lysates, (F) target engagement using CETSA-western blot for PFKM, RIA, RIIB and actin, and (G) lactate secretion, glucose uptake, intracellular lactate and glucose levels. RLU: relative luminescence units. * <0.05, **, <0.01, two-tailed t-test.

**Fig 3:**
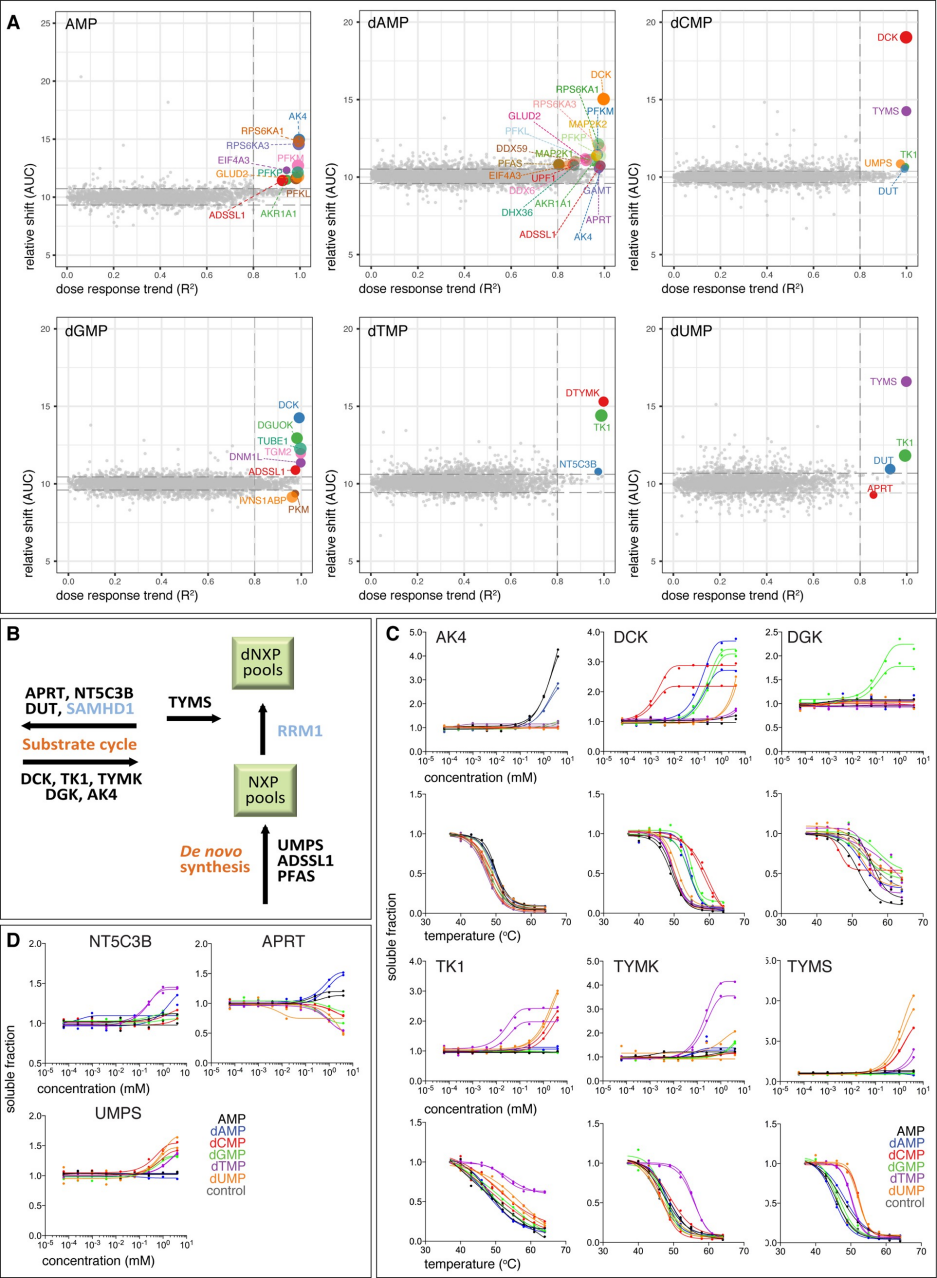
Protein hits from lysate CETSA with deoxynucleotides. (A) ITDR_CETSA_ hit plot with AUC versus R^2^ plot of ITDR_CETSA_ curves after lysate treatment with AMP, dAMP, dCMP, dGMP, dTMP, and dUMP. Each dot represents the average AUC (relative thermal shift) and R^2^ ITDR_CETSA_ (dose response trend) measurements per protein from two replicates. The size of the dot negatively correlates with the MDT. (B) Schematic of the proteins in the core nucleotide metabolism responding to the five deoxynucleotides and AMP. SAMHD1 and RRM1 were hits in the in-cell ITDR_CETSA_ experiment (Fig 5). (C) ITDR_CETSA_ (top) and CETSA curves (bottom) with the dNMPs for enzymes involved in the deoxypyrimidine salvage and synthesis pathways; adenylate kinase 4 (AK4), deoxycytidine kinase (DCK), deoxyguanosine kinase (DGK), thymidine kinase (TK1), thymidylate kinase (TYMK) and thymidylate synthase (TYMS). Data is presented as two individual technical replicates for each condition from one representative experiment. (D) Other protein hits from dNMPs-ITDR_CETSA_ involved in the nucleotide metabolism: 7-methylguanosine specific 5’-nucleotidase (NT5C3B), adenine phosphoribosyltransferase (APRT) and UMP synthase (UMPS). Data is presented as two individual technical replicates for each condition from one representative experiment.

**Fig 4:**
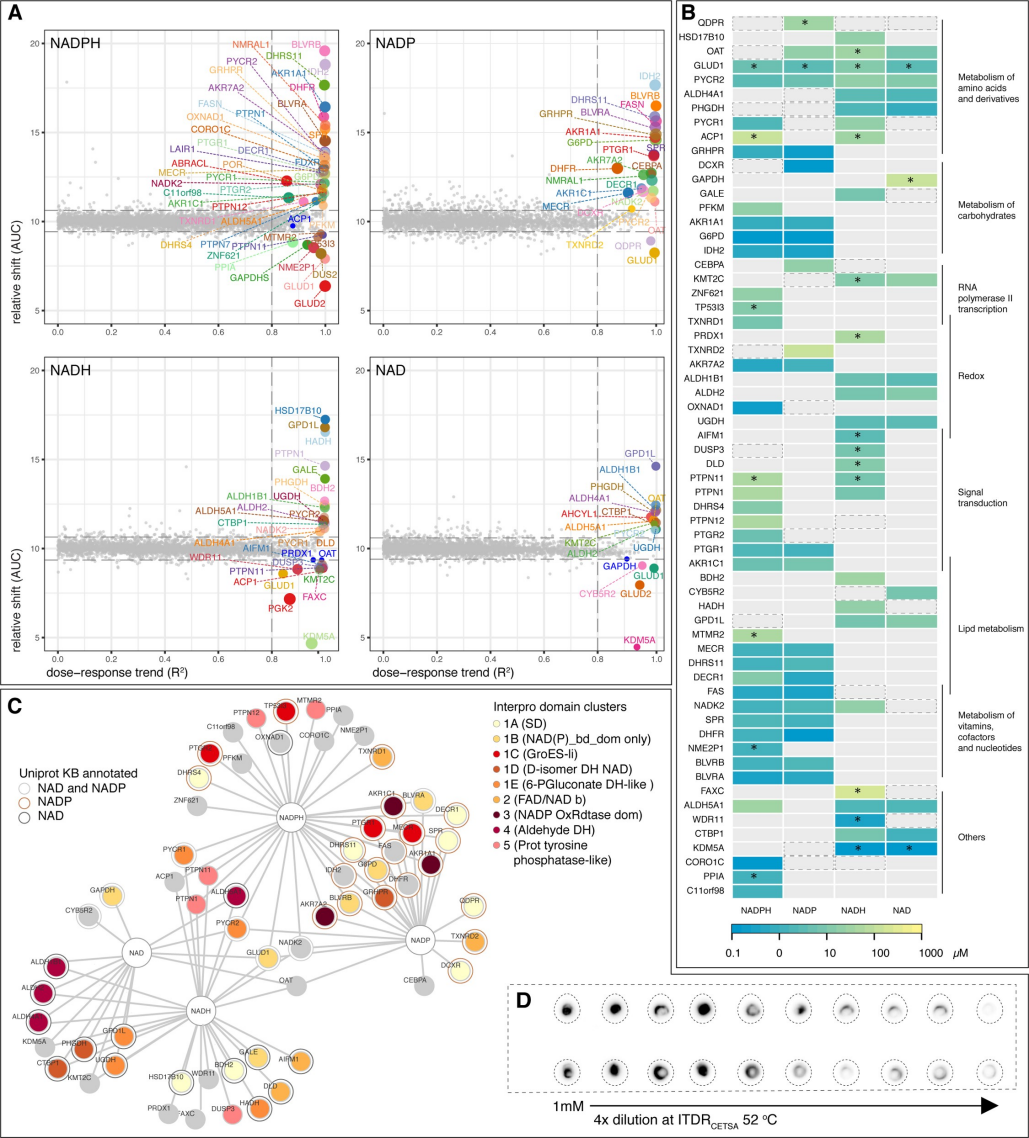
Protein hits from NAD(P)(H) ITDR_CETSA_. (A) ITDR hit plots with AUC versus R^2^ plot of ITDR_CETSA_ curves from lysates treated with NADPH, NADP, NADH, and NAD. Each dot represents the average AUC (relative thermal shift) and R^2^ ITDR_CETSA_ (dose response trend) measurements per protein from two replicates. The size of the dot negatively correlates with the MDT. (B) Relative affinities and specificities of the four nicotinamide-based nucleotides for their identified hit proteins. Colour scale represents the minimal dose threshold (MDT in μM) of the isothermal dose response at 52°C, with the lowest MDT (therefore higher relative affinity) in blue and the highest MDT (therefore lower relative affinity) in yellow. Hit proteins are grouped according to their associated biochemical pathways and function (black lines), and overlaps indicate their association with more than one pathway/function. Asterisks (*) indicate destabilizing hits. Black dotted boxes indicate proteins with similar ITDR responses that did not meet the hit selection criteria. (C) Protein domain analysis in the NAD(P)(H) hit proteins. Hit proteins are clustered in 5 broad Interpro domain clusters (1–5), with 5 subclusters (A-E) under cluster 1. NAD annotated protein (black circular outline), NADP (red circular outline), NAD and NADP annotated (grey circular outline). Unannotated proteins are not outlined. Annotations were taken from Uniprot KB. (D) Thermal precipitation assay with recombinant CORO1C and increasing doses of NADPH at ITDR_CETSA_ 52°C.

We used the isobaric tagging-based multiplexed quantitative mass spectrometry (MS) method introduced for CETSA experiments on drug binding by Savitski et al. [18, 23] but established a MS-CETSA protocol with a distinct data processing and analysis strategy to allow for sensitive and stringent hit generation. This was enabled by a novel versatile R package (“mineCETSA”) that was developed in-house for systematic data scaling, analysis and visualization (S1A Fig). Melting curves were previously used for selecting hit proteins from MS-CETSA data [18], so we first collected replicate CETSA melting curves (37 to 64°C, 3°C interval) from non-treated K562 lysates or lysates treated with each of the eight nucleotides (AMP, dAMP, dCMP, dGMP, dTMP, dUMP, cAMP, cGMP). We collected in parallel the so-called isothermal dose response data (ITDR_CETSA_) for these nucleotides at 52°C, which is the median temperature of the K562 soluble proteome. We noted that the ITDR_CETSA_ was much more distinct in revealing known metabolite interactions than melting curves, which is likely due to the multiplexing of control versus treated data points being within the same isobaric tagging-based multiplex in the ITDR_CETSA_ experiment. Stronger responders, however, still had corresponding CETSA shifts in the melt curves (Figs 2 and 3). We therefore developed a novel computation strategy for the ITDR_CETSA_-based hit generation from this data based on a variance analysis for each individual protein (AUC versus R^2^ plots in Figs 2B, 3A and 4A) to visualize the hits against the proteome. In addition to being more robust for hit selection, ITDR_CETSA_-based hit generation also has the advantage of containing information on relative affinities [16, 24, 25]. We subsequently quantified interactions with the eight nucleotides for a total of 7234 proteins (4000–5000 proteins per nucleotide) and detected a total of 46 protein hits that interact with at least one of the eight nucleotides based on their significant stabilization/destabilization effects (S1 Table). We also performed controls at 37°C for these nucleotides to confirm that no hit-protein had changed protein levels during the nucleotide incubation time (S11 Table). The heatmap in Fig 1B shows the distribution of the hit proteins that were detected in all eight nucleotides into different pathways/classes, so as to illustrate the nucleotide binding specificity of these proteins. The intensity of the colors indicates the minimal dose threshold (MDT), which is the lowest concentration that gives a measurable stabilizing (or destabilizing) effect at the specified CETSA heat temperature for the nucleotide and its hit protein. It is evident from this map that the nucleotide CETSA interactions are highly specific and although most of the interactions are already described, we can also find some novel interactors with the CETSA approach (S2 Table). In general, most metabolites have a stabilizing effect on the protein but there were also exceptions. For example, cAMP was found to significantly stabilize 10 proteins and destabilize 2 proteins (Fig 2B and S1 Plot). In the processing of several initial datasets, we found a smaller group of proteins that were sensitive to small pH changes (S2 Plot and S3 Table). We therefore introduced more controlled buffer conditions, and in subsequent experiments presented in our analysis, we did not see concerted shifts in these proteins (S3 Plot).

### MS-CETSA identifies known and novel targets of nucleotide-based second messengers

cAMP and cGMP are nucleotide-based second messengers that typically act downstream of G-protein coupled receptors (GPCRs) whereby cyclic nucleotide-dependent protein kinases, as well as proteins from other pathways are activated [26–30]. We first assayed the concentration of cAMP as a representative nucleotide in the lysates for up to 24 hrs (S1B Fig) to ensure that cAMP was still available for protein binding. The common hits in the ITDR_CETSA_ (and melt curve shifts) for both cAMP and cGMP in K562 lysates include subunits of cAMP-dependent protein kinase A (PKA) (Fig 2A). The cGMP-dependent kinase, however, is generally expressed at low levels, hence it was not detected in the present experiment [31]. The four regulatory PKA subunits (PRKAR1A or RIα, PRKAR1B or RIβ, PRKAR2A or RIIα, PRKAR2B or RIIβ) showed strong stabilization by cAMP in ITDR_CETSA_. The catalytic subunit α (PRKACA or Cα) on the other hand displayed a negative shift that is likely due to loss of interaction with the regulatory subunit, as was previously suggested [25]. In the case of PKA subunits, the MDTs in general affirmed specificities reported by the relative rankings of the affinities of the regulatory subunits, with RIs having lower MDTs than RIIs for cAMP [32, 33]. The PKA subunits mostly responded at a higher concentration of cGMP as compared to cAMP, except for the the RIIβ subunit. Interestingly, RIIβ was destabilized by cGMP throughout the concentration range (Fig 2A) and also responded earlier to cGMP than the other regulatory subunits. This is unexpected and the potential mechanistic implications are outlined in S1C Fig.

Another prominent hit related to the PKA system is the small membrane A-kinase anchor protein (SMAKA) (Fig 2C). It was first identified by cAMP-based chemical proteomics as a RI-specific PKA-anchoring protein (AKAP) [34], but we found that it also responded to cGMP (Fig 2C). However, a direct interaction between the cyclic nucleotides and SMAKA is less likely and therefore the observed stabilization might be due to secondary effects involving other protein components instead, as outlined in S1C Fig [35]. Although interactions of cAMP and cGMP analogs with the human proteome have previously been studied using chemical proteomics, the present data gives, arguably, a more direct view of the specificities of the interactions of these second messengers with PKA components. There are however a number of additional known membrane-bound PKA binding proteins of the A-kinase anchor protein family that were successfully identified in chemical proteomics experiments [11, 36] but were not detected in our experiments as detergent was not present in the CETSA protocol.

In addition to the PKA system, there were nine hits that passed our selection criteria (Fig 2B and S1 Plot and S4 Table). The three hits with the lowest MDTs in ITDR_CETSA_ for cAMP were different homologs of phosphofructokinase (PFK) (Fig 2D). PFK is the rate-limiting step in glycolysis and integrates multiple metabolic signals through its allosteric ligands such as AMP, ATP and fructose 2,6-bisphosphate. Although cAMP is relatively less studied, it has been reported as a regulatory ligand for rabbit skeletal muscle PFK [37]. Our CETSA data supports that the homologous platelet and liver PFK bind to cAMP with similar affinities and are likely to be regulated by this second messenger. The six additional hits for cAMP and cGMP have higher MDTs and are mainly known nucleotide interacting proteins (Fig 2B and S1 Plot). Although these are candidates for relevant interactions at physiological metabolite concentrations, several of these might correspond to low-affinity background binding due to similar chemical structures to other purine nucleotides (discussed below). Together, this supports a low rate of false positives for our new CETSA protocol for these nucleotides. In a curated search for cAMP and cGMP binding proteins in our dataset, we only find cAMP and cGMP phosphodiesterases to be missing in our hit list, although several of them start to melt well before 52°C and could be expected to respond in the ITDR_CETSA_ experiment. However, the cAMP and cGMP phosphodiesterases present in our CETSA datasets are either assigned as membrane proteins (PDE4A/PDE6D/ PDE3A/PDE9A), or are found to be toggling between membranes and the cytoplasm (PDE11A), which might explain the lack of response for these proteins in the detergent free conditions used in the present study.

To gain further insights into intracellular cAMP binding, and given cAMP’s role in regulating glucose metabolism [38], we stimulated cAMP production in cells and monitored changes in glucose and lactate levels along with target engagement of the PKA system and PFKM. We treated intact K562 cells with forskolin, an adenylyl cyclase activator and a GLUT transporter inhibitor [39, 40], and measured intracellular cAMP as well as target engagement for PFKM, RIA and RIIB with western blot-based CETSA. Intracellular cAMP levels were studied at 0, 15 and 60 min as well as overnight, which peaked at 60 min (Fig 2E). RIA showed clear target engagement saturation from 15 min onwards whereas RIIB did not show target engagement at these conditions (Fig 2F). Judging from the lysate ITDR experiments, this can be explained by the 70-fold higher cAMP concentration required to stabilize RIIB than for RIA (Fig 2A and S4 Table). PFKM, which also responds at similar concentrations as RIIB in the lysate, did not shift in the cells at the conditions used for the CETSA experiment (Fig 2F). We observed significant reduction in lactate secretion and glucose uptake with no significant intracellular lactate and glucose changes upon overnight incubation (Fig 2G).

We also studied target engagement of the same set of proteins using cell permeable cAMP, bucladesine (Db-cAMP) and 8-bromo-cAMP (Br-cAMP). We treated cells with 2 mM of each analog, however we were only able to measure the intracellular concentration of Db-cAMP (Fig 2E) but not the Br-cAMP. The latter is likely due to the bromo-substitution sterically interfering with the anti-cAMP antibodies’ ability to capture the modified cAMP in the assay. For both cAMP analogs we observed target engagement of RIA, which supported their intracellular uptake and binding (Fig 2F). Similar to forskolin-induced cAMP, we did not see engagement of the two analogs with PFKM and RIIB in cells (Fig 2F). We observed however, a significant increase in intracellular lactate and a decreasing trend in glucose uptake only under Db-cAMP-treated conditions and not for Br-cAMP (Fig 2G). This supports Db-cAMP’s upregulation of lactate dehydrogenase’s activity and inhibition of GLUT transporter [41, 42]. Together we show that cAMP and the two analogs can activate PKA through RIA but that they exert distinct effects on glucose metabolism. Due to the known pleotropic effects of cAMP on the glucose metabolism[38], the data does not however allow for any direct conclusions on whether cAMP can play a role or not as a PFK regulator.

### Specificity in dNMP deoxynucleotide synthesis and salvage pathways

(Deoxy)nucleotide monophosphates ((d)NMPs) are generated through *de novo* synthesis or salvage of circulating nucleosides and nucleobases. Enzymes of the human (d)NMP metabolism are targets for many drugs in clinical use and mutations in these pathways lead to distortions in nucleotide pools and cause several inborn diseases [43]. Examples of key proteins are nucleoside kinases, which play a role in the salvage of (d)NMPs and nucleotidases, which play the opposing role of hydrolyzing (d)NMPs to their corresponding nucleosides.

We performed MS-CETSA shifts and ITDR_CETSA_ experiments for dGMP, dAMP, dCMP, dUMP, and dTMP in K562 lysates (Fig 3 and S4 Plot and S5 Table). ITDR_CETSA_ hits from AMP were included to indicate background binding to ATP binding sites. 34 proteins passed our hit-selection criteria as potential dNMP-interacting proteins (Fig 3A and S4 Plot). Analysis of the human *de novo* and salvage nucleotide metabolism pathways (Fig 3B) demonstrated that a significant number of the proteins in these pathways, 12 proteins, gave an ITDR_CETSA_ response with at least one nucleotide (Fig 3B and S4 Plot). Six of the 10 hit proteins from the AMP data set are known ATP binders such as kinases and ATPases (Fig 3A and S4 Plot). However, many of these have high MDTs, which could reflect background binding due to similar chemical structure to ATP/ADP. Several known GXP binding proteins, TUBE1, TGM2, DNM1L, also responded specifically to dGMP at high MDTs (Fig 3A).

A subset of the most prominent hits for dNMPs are enzymes in deoxynucleotide salvage and synthesis: adenylate kinase 4 (AK4), deoxycytidine kinase (DCK), deoxyguanosine kinase (DGK), thymidine kinase 1 (TK1), thymidylate kinase (TYMK), and thymidylate synthase (TYMS) (Fig 3C). Interestingly, the dNMPs are either substrates or products for these enzymes, supporting that CETSA can yield valuable information on both substrate specificity and susceptibility to product inhibition. For example, DCK is known to have deoxycytidine as its primary substrate but it also phosphorylates deoxyadenosine and deoxyguanine [44], forming dAMP and dGMP respectively. To further confirm the detection of substrate and product interactions with the thermal shift approach, we purified DCK and performed thermal shift assays with a range of nucleoside-based compounds (S6 Table). This data is consistent with the ranking observed in the lysate CETSA experiment and reveals the characteristic specificity of DCK for dNMPs (S6 Table).

A number of interactions with other enzymes in the human nucleotide metabolism with potential novel implications for their functions were also found (Fig 3D). UMP synthase (UMPS), a bifunctional enzyme in the *de novo* synthesis of UMP interacted at higher MDTs with all dNMPs except dAMP. We purified the two catalytic domains of UMP synthase individually and confirmed that these dNMPs (as well as several NMPs) stabilized the orotidine-5’-phosphate decarboxylase domain (S6 Table). This opens up the possibility that UMP synthesis is regulated by (d)NMP pools at higher concentrations. The 7-methylguanosine specific 5’-nucleotidase (NT5C3B) made an unanticipated interaction with dTMP, which suggests that it has an extended specificity than previously reported [45]. Together, this supports that CETSA can detect interactions with a range of different dNMP binding protein families, where most interactions constitute substrate and product interactions.

In the hit list for dNMPs in Fig 1B, all proteins are either known dNMP binders or are known to bind other related nucleotides. This suggests that the identified interactions are real and that rates of false positives are very small. Considering the other side of the coin i.e. non-responders, only 9 proteins remain that do not respond in our measured data set, out of a curated list of proteins that are known to interact with dNMPs. These are nucleotide phosphatases, nucleotide kinases and a nucleotide deaminase and out of these, 6 proteins (NT5C2, NT5C, dCTP1, DCTD, TK2 and AK2) start melting at high temperatures and would not be expected to respond in a 52°C ITDR_CETSA_. The remaining three are kinases (CMPK1/2 and AK1). These might be non-responsive in the CETSA assay, or alternatively, they could be present in the lysate in a state where dNMP would not bind. Such situations could appear, for example, if ATP binding is required to pre-organize the active site for the binding of the co-substrate dNMP and the ATP concentration in the lysate is not sufficient to accomplish this.

We were intrigued by the stabilizing interactions of dCMP with TYMS and deoxyuridine 5’-triphosphate nucleotidohydrolase (DUT) (Fig 3A and 3C and S4 Plot), as dCMP is not expected to interact with these well-characterized proteins. We confirmed with western blot-CETSA that dCMP and dUMP had similar MDTs with TYMS as in the MS data (S1D Fig). We also measured dCMP’s target engagement with a recombinant truncated TYMS variant and its effect on the enzymatic activity. We observed a thermal shift with dCMP (S1E Fig), and inhibition of the activity of the recombinant protein, albeit at a much higher effective concentration (EC_50_) than dTMP (S1F Fig). We subsequently determined the crystal structure of dCMP with TYMS, confirming binding in the active site (S1G Fig). Due to this discrepancy in affinities, it is a possibility that dCMP was metabolized to dUMP in the lysate. To shed further light on the stability of dCMP and other dNMPs in the lysate experiment, we used alphascreen-CETSA to monitor target engagement of lysate TYMS with dUMP, dTMP and dCMP from 15 sec to 10 min (S1H Fig) [46]. No time dependency was seen for the ITDRs with dUMP and dTMP in the lysate, supporting the inertness of these nucleotides during the period of the experiment (3 min). In the dCMP ITDRs, however, there was a strong time dependency whereby target engagement was low at 15 sec but increased over time. The MDT was also much higher at 3 min as compared to the MS- and western blot-CETSA of the same duration. Based on these results we hypothesize that there are two possible forms of TYMS in the lysate. One form is similar to the purified TYMS (S1E and S1F Fig) and is specifically recognized by the pair of antibodies from the alphascreen (S1H Fig). This form shows much lower affinity for dCMP than for dUMP and the shift observed in the alphascreen is due to the deamination of dCMP to dUMP by dCMP deaminase in the lysate. The MS- and western blot-CETSA (Figs 3C and S1D) however, detects another form of TYMS that binds unmetabolized dCMP within physiological concentration. The exact role for this interaction however, remains uncertain. Together this suggests that CETSA (and TSA) studies in multiple formats, combined with time dependency studies, allows for more resolved information on the interactions to be obtained. Since the binding of metabolites to proteins in most cases is expected to be rapid, the usage of shorter incubation times than presently applied, might be merited for future studies.

### Specificities of NAD(P)(H) binding proteins

The nicotinamide adenine dinucleotide coenzymes NAD(P) are predicted to interact with a relatively large number of proteins. However, the majority of such interactions with human proteins have never been experimentally determined but are only assigned based on their homology to related proteins (families) that have NAD(P)(H) as an experimentally confirmed ligand. Therefore, to confirm predicted interactions with the human proteome and to discover novel ones, we collected ITDR_CETSA_ for NADPH, NADP, NADH and NAD, which yielded a hit list of 73 proteins (Fig 4A and S1, S2 and S7 Tables). Of this list, 47 proteins were experimentally validated or predicted target proteins for their respective CETSA ligand hits (S7 Table). We added 1mM NADPH to lysates and measured its availability over 24 hrs and found that there were constant amounts of NADPH up till at least an hour of incubation (S2A Fig).

We generated a heatmap of ITDR hits from the subset of 62 proteins that were detected in all 4 experiments (Fig 4B), termed as the complete subset, to have an overview of the specificity of the ITDR responses. 44 proteins from this subset have previously been annotated as NAD(P) binding proteins (S7 Table). These NAD(P)(H) binding proteins, mostly dehydrogenases and reductases, cover a range of cellular pathways and localizations and gave MDT responses at relatively narrow concentration ranges, which might correlate with the intracellular NADPH concentration (Fig 4B and S5 Plot). 19 proteins from this subset have yet to be annotated as NAD(P) binding proteins for their respective hit ligands thus far (S7 Table).

We aligned the complete subset based on their Interpro domain notations (Figs 4C and S2B) and found that the proteins formed 5 major clusters (clusters 1 to 5) according to their domains and superfamilies. Cluster 1 (A to E) was the largest cluster constituting the NAD(P) binding domain superfamily. The proteins in the subclusters showed predominant specificities for either NADP(H) or NAD(H), with five exceptions (QDPR, GLUD1, PHGDH, PYCR1, PYCR2) that interacted with all four forms. The four proteins in cluster 2, including the two thioredoxin reductases, had specificities for either NADP(H) or NAD(H). The specificities of the proteins in cluster 3 and 4 were primarily towards NADP(H) and NAD(H) respectively. Cluster 5, however, did not contain NAD(P) binding domains and is constituted by 5 phosphotyrosine phosphatases (PTPases) family members: PTP1B (also known as PTPN1), PTPN12, PTN11, DUSP3 and MTMR2. Interestingly, these proteins were shifted only by the reduced nucleotides NADPH and NADH (Fig 4B). We hypothesize that these stability changes were due to the oxygenation of active site cysteine residues of these PTPs by reactive oxygen species (ROS) generated from NADPH binding proteins (e.g. NADPH oxidase). Another protein-tyrosine phosphatase, ACP1 (LMW-PTP), which has similar active site architecture, was also shifted by the reduced nucleotides. Oxidation of the conserved active site cysteine residues is a well-established regulatory mechanism of the activity for PTP1B, PTPN12, and LMW-PTP [47, 48]. Six known NAD(P) proteins that do not belong to the 5 clusters, i.e. FAS, IDH2, NADK2, OXNAD1, CYBR2 and DHFR, were identified with their expected specificities for the NAD(P)(H) ligands. Out of the remaining 13 unannotated hit proteins, shifts for 7 proteins (KDM5A, KMT2C, FAXC WDR11, CEBPA OAT, CORO1C) are more likely to be caused by direct NAD(P) interactions, rather than ROS modifications, when they respond to both oxidized and reduced nucleotides. Out of these 7 proteins, 6 only shift at high MDTs or give negative shifts. However, one protein, coronin 1c (CORO1C), gave a strong response in the expected physiological range for NADPH, as well as a weaker response for NADP+ within this range suggesting that this is a candidate for a novel direct NADPH interaction (Fig 4A and 4B). Coronins belong to a family of ubiquitously expressed proteins of which some have been shown to play roles as actin regulators. Coronin 1c has been proposed to interact with subunits of NADP oxidase [49]—this enzyme uses NADPH to generate superoxide and thereby plays an important role in fighting against microbial infections and in the pathology of atherosclerosis [50]. The CETSA effect on coronin 1c induced by NADPH and NADP+ suggests a previously unknown interaction and redox role for NADPH with this protein. We verified the direct effect of NADPH on the thermostability of recombinant coronin 1c using thermal precipitation assay (Fig 4D).

A key unanswered question related to the usefulness of CETSA for proteome-wide studies is the rate of false negatives, i.e. proteins that do not show a CETSA response upon cognate ligand binding. Considering the relative large numbers of hits for NADPH we decided to use this compound as a reference to estimate false negative rates. As discussed above, the 52°C ITDR_CETSA_ will not yield shifts on proteins with melting curves starting above 52°C. Hence, we performed an additional ITDR_CETSA_ experiment for NADPH at 58°C (S6 Plot and S8 Table) and identified other potential NADP-binding proteins (S6 Plot). We subsequently used an unbiased NADPH prediction and made an assessment for how many known NADPH binding proteins can be considered true non-responders in CETSA (S2D Fig and S6 and S7 Plots and S9 Table). Excluding proteins with extremely high melting temperatures (S7 Plot), which might need ITDRs at even higher temperature than 58°C, only a few proteins did not show shifts, suggesting that the non-responding rates could be as low as 10% (S9 Table). The estimated low rate of false positives and negatives in these experiments is likely due to the stringent ITDR_CETSA_-based strategy for hit generation.

### In-cell MS-CETSA identifies a cascade of metabolite-protein interactions following thymidine import

After demonstrating that the ITDR_CETSA_ hit selection approach is a robust strategy for establishing specificities of nucleotides in lysate experiments, we performed in-cell MS-CETSA of intact K562 cells exposed to thymidine (dT). The cell cycle arrest at the G1-S transition is a well-known effect of exposing cells to mM levels of dT. This phenomenon has been explored in the widely used double thymidine block (DTB) for cell synchronization [51, 52]. We first focused our in-cell CETSA studies on the effects of thymidine exposure for 3 hours. Exposure to high levels of dT for 3 hours yielded no significant difference in the distribution of cells between the different phases of cell cycle, while exposure for 24 hours changed the distribution significantly towards G1-S phase as expected (S3A–S3C Fig). Similar to the lysate experiments, we used ITDR_CETSA_ for the in-cell experiment, and in addition investigated several experimental approaches involving different conditions and sequences for cell lysis, cell washing and heating. The final data was collected as two biological replicates, each with two technical replicates (S3D Fig). To confirm that we have not changed protein levels during the incubation, we also monitored changes in protein levels during this period where only a few proteins showed significant alterations of protein levels (S3E Fig).

**Fig 5:**
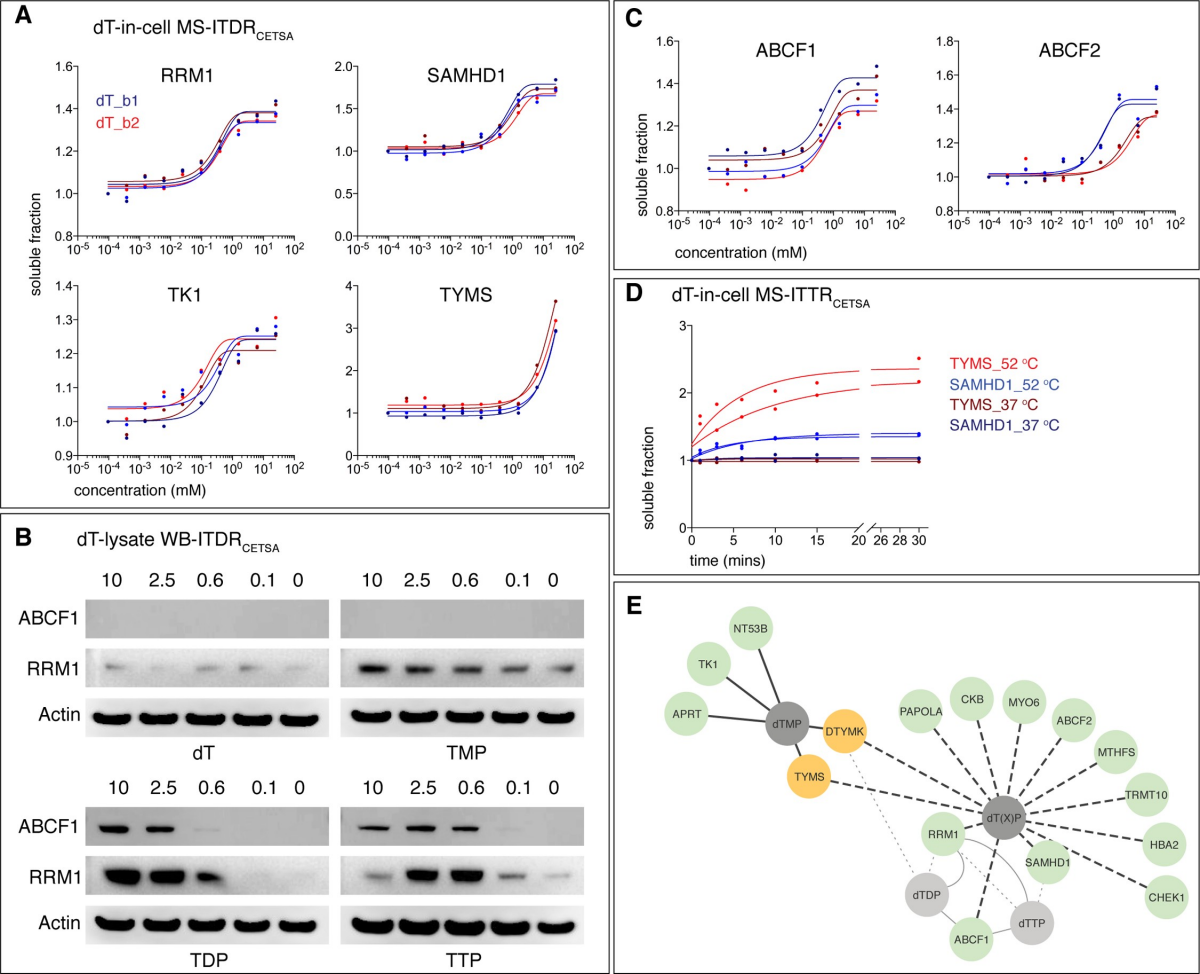
Hit proteins from the in-cell ITDR_CETSA_ experiments with thymidine. (A) In-cell MS-ITDR_CETSA_ curves of enzymes involved in the thymidine metabolism cascade. (B) Lysate-western blot (WB)-ITDR_CETSA_ validation with dTXP. Lysates were treated with 10, 2.5, 0.6, 0.1 and 0 (control) mM of dT, TMP, TDP or TTP and probed for RRM1 and ABCF1 with actin as loading control. (C) In-cell MS-ITDR_CETSA_ curves of ABCF1 and ABCF2. Data is presented as two individual technical replicates for each condition from 2 biological replicates (biological replicate 1 (dT_b1, blue) and biological replicate 2 (dT_b2, red)). (D) Thermal stabilization kinetics between TYMS (red) and SAMHD1 (blue) from the in-cell ITTR_CETSA_ experiments with thymidine. Data is presented as two biological replicates at 37°C (TYMS_37°C, SAMHD1_37°C) and 52°C (TYMS_52°C, SAMHD1_52°C), with focus on the incubation duration of 0 to 30 mins. (E) Summary of hits identified by: in-cell MS-ITDR_CETSA_ (dotted lines); lysate MS-ITDR_CETSA_ (solid lines); validated by WB-ITDR_CETSA_ (grey dotted lines). Common hits from the in-cell and lysate MS-ITDR_CETSA_ (yellow circle); metabolites (grey circle); known metabolite-protein interactions (grey solid lines).

Using a similar scoring scheme as developed for the lysate experiments, we established a primary hit list of 13 proteins (S8 Plot). Four of these are well-characterized enzymes either involved in deoxynucleotide salvage and synthesis pathway (Fig 5A). These proteins are RRM1, SAMHD1, TK1, and at higher MDT, TYMS [53–56]. In the case of TK1 it could be either, or both, substrate (dTMP) or/and product (dTDP) that stabilize. The observed response for TYMS is likely due to the binding of dTMP as a product, as shown in our deoxy-lysate ITDRs discussed above, and shows that CETSA can be used to access product binding/inhibition in cells. The other hits in the deoxyribonucleotide synthesis were RRM1, the catalytic subunit of ribonucleotide reductase (RNR), the enzyme that converts NDPs to dNDPs and is allosterically regulated by dTTP [57], and SAMHD1, the dNTP hydrolase regulated by dNTPs that has dTTP and dT as substrate and product respectively [58, 59]. We confirmed with western blots-based CETSA the binding of RRM1 (a key allosteric regulator of RNR) to dTTP in lysates (Fig 5B). In addition, we observed binding of RRM1 to dTDP but neither to thymidine nor dTMP (Fig 5B). This observation was also affirmed by TSA of purified RRM1 (S6 Table). This supports that RNR interacts with, and is likely regulated by dTDP which constitutes a novel regulatory mechanism for RNR [57]. Among the remaining 9 hits, only 5-formyl tetrahydrofolate cyclo-ligase is known to be connected to deoxyribonucleotide metabolism though its involvement in the pathways generating 5,10-methylenetetrahydrofolate, the substrate for TYMS.

Most of the additional hits are proteins that appear to have nucleotide binding sites and/or are involved in RNA-processing pathways. We were intrigued by the presence of the two homologous ATP-binding cassette sub-family F members 1 and 2 (ABCF1 and ABCF2) in the hit list (Fig 5C). ABCF1 and ABCF2 are soluble proteins containing the ATP binding moieties of ABC transporters. We confirmed using western blotting in lysates that dTTP and dTDP, but not dTMP or thymidine, indeed stabilized ABCF1 (Fig 5B). We subsequently purified the full length ABCF1 to evaluate potential interactions and showed that both dTTP and dTDP make direct interactions apparently with similar affinities as ATP and ADP (S3F and S3G Fig). The role of these interactions with ABCF1 remains speculative at this point but possibilities are that dTTP hydrolysis serves to drive specific structural changes under certain conditions, or that ABCF1 serves as a phosphatase, scavenging dTTP in certain cell states [60]. The latter would be similar to that has been proposed for SAMHD1, which after activation by phosphorylation, depletes cells of certain (d)NTPs. For the remaining hits, we did not try to confirm whether they make direct interactions to thymidine-based nucleotides, so it remains possible that a number of these shifts are due to downstream indirect effects of the thymidine treatment.

Many cellular processes are sequential in nature and we implemented a time dependent CETSA protocol for studies of metabolite-protein interactions, which allowed us to follow the uptake and conversion of thymidine into its phosphorylated products (dTMP, dTDP, and/or dTTP). This was done by incubating the cells at a constant dose and tracking the proteome-wide CETSA response over time; we term this approach isothermal time response (ITTR). The ITTRs were performed at 52°C and also 37°C; the latter to track changes in protein levels in the cells during the incubation period. Although several of the aforementioned dT hits in the cell were not detected in the MS experiment, binding to TYMS (target of dTMP) and SAMHD1 (target of dTTP) showed ITTR responses, both reaching half saturation already after a few minutes treatment (Fig 5D). This supports a rapid uptake and subsequent phosphorylation of thymidine.

Together the in-cell dT experiment support the robustness of our experimental and hit selection procedures, and that MS-CETSA will be a very powerful approach to discover and map intracellular metabolite-protein interactions that have previously been inaccessible. The study also indicates that the combination of dose- and time-dependent experiments can, potentially, give very high-resolution insights into cellular processing of metabolites. We also found effects on proteins outside the core thymidine metabolism in the in-cell experiments. These could be the downstream effects from the generation of thymidine metabolites and therefore constitute candidates for involvement in the still poorly characterized mechanism for the double thymidine block.

## Discussion

Here we introduce a stringent strategy for unbiased discovery and characterization of novel protein-metabolite interactions with CETSA and apply it to nucleotide-based metabolite interactions with the human proteome. Using ITDRs, rather than melting curves as the primary means for hit generation, we confirmed that CETSA detected many known interactions that were previously established with traditional biochemistry over several decades. We could also experimentally confirm many interactions for NAD(P)H that were previously only predicted based on homology and determine their specificities for the four structural and redox forms of these nucleotides. For all nucleotides we revealed novel candidates for cognate interactions and verified several of these with studies on purified proteins. We detect regulatory interactions such as for RNR and the PKA system, but also many interactions with substrate and products of enzyme reactions. As discussed above, the analysis of the data suggests a low rate of false positives. The analysis also supports the potential for low rate of false negatives, as illustrated by the NADP(H) data, although there is likely to be protein types/families and proteins in certain complexes that are less responsive to CETSA.

The parallel profiling of nucleotides that share similar chemical structures allow us to rank interactions for each target protein, and therefore assess the binding specificity of these proteins. In instances where the interaction was only observed at the highest concentration, these interactions might reflect background binding due to similar chemical structures to the higher affinity cognate nucleotide. These said metabolite-protein interactions should ideally be referenced against the intracellular concentrations of metabolites, or when feasible, confirmed with a CETSA experiment on intact cells.

The human cell has thousands of metabolites for which corresponding protein interactions remain mostly uncharacterized. CETSA therefore offers the advantage of a generic strategy for stringent identification of novel targets and specificities and should be applicable to a wide range of metabolites. In cell lysates, CETSA circumvents the need to develop affinity or reactive probes, which can be a challenging endeavor. Although different affinity proteomics approaches have been successfully applied for identifying drug targets [61], such approaches have only been applied to studies of a limited number of metabolite interactions, i.e. certain nucleotides (NTPs [62]^,^ [63]) and cNMPs [12]), lipids and cholesterol [13, 14]. As for CETSA, the recently introduced LiP-MS method provides a potential method for label-free discovery of novel metabolite interactions in lysates, but cannot, in contrast to CETSA, be applied to intact cells. The comparison of these two label-free methods on the same lysate samples would be very interesting to assess their relative properties.

The in-cell CETSA metabolite experiments are particularly exciting, as proteins and their associated complexes are not perturbed by cell lysis during treatment and are found in their natural locii. The experiment also integrates transport events of the precursor metabolite and allows studies of interactions made by a cascade of metabolites subsequently formed within the cell. This is illustrated by our thymidine experiment, where upon internalization, thymidine is phosphorylated to dTMP, dTDP and dTTP and yielded interactions with an ensemble of proteins (Fig 5E). The possibility of adding a time dependency to the interactions, as illustrated for thymidine above, adds another important dimension to resolving the sequential activation of metabolite-regulated nodes. The status of such nodes is important in defining the metabolic state of cells and CETSA-responsive proteins could therefore find roles as biomarkers for specific cellular metabolic states for use in research and clinical diagnostics. In a very recent report from our group that uses CETSA to study the cell cycle, several of the thymidine shifting proteins indeed gave negative shifts after the release from a double thymidine block [64].

CETSA might also help in revealing key enzymes and regulatory nodes in metabolic pathways that can serve as therapeutic targets. This was illustrated by thymidine, which is limiting for cancer cell proliferation. In the in-cell CETSA experiment with thymidine, two important drug targets for cancer therapy were directly revealed; TYMS targeted by e.g. fluorouracil and diverse antifolates [65], and RNR, targeted by e.g. clofarabine and gemcitabine [66]. For less well-studied metabolites that are limiting for cancer growth, CETSA should be an ideal strategy for discovering novel protein candidates as drug targets. When developing a drug that targets metabolic enzymes, it is often challenging to establish a cellular assay for high-throughput screening (HTS) and for ligand optimization. We have recently showed that CETSA is an efficient HTS assay for cellular target engagement [46] and it should be particularly useful in aiding the process of generating cell active inhibitors against metabolic enzymes. Here we report more than 100 metabolic enzymes that are responsive in CETSA, for which cellular target engagement assays using CETSA should be feasible.

As discussed above, we introduced the use of ITDRs for hit selection in proteome-wide CETSA experiments, which is a more distinct strategy compared to the previous approach based on CETSA shifts. Importantly, the ITDR-based strategy also allows the detection and relative ranking of weak metabolite-protein interactions. The multiplexing of all 10 conditions used for ITDRs and ITTRs in the same MS run reduced the inter-condition variances contributed by separate MS runs, which was observed in previous melting curve experiments. In the initial experiment we used the median proteome melting temperature of the cell line K562 (52°C) for our ITDRs, thus potentially missing shifts of some proteins that unfold at either higher or lower melting temperatures. With an additional ITDR at 58°C for NADPH, we improved the number of hits for cognate NADP-binding proteins by 40%. For a guaranteed coverage of the proteome, ITDRs at further temperatures might be needed. Recently, Savitski et al. presented a strategy using 12 different temperatures and five concentrations of a drug [24]. This two-dimensional approach also significantly improved stringency of hits selection as compared to previous melting curve-based strategies, but do not specifically address the challenge of detecting and ranking both strong and weak ligands[24]. We anticipate that a strategy that combines our approach, which has a more extensive dose sampling for an improved ranking of compound MDT, improved coverage of the affinity range and individual error estimates, with multiple temperature ITDR measurements, would be ideal for studies of metabolites and other lower affinity interactions in cells.

## Supporting information

S1 Fig Supporting data for cyclic and deoxynucleotide ITDRs. (A) Hit selection scheme in ITDR_CETSA_ experiment. The readings from the lowest three concentration groups are used to derive an upper and lower baseline variance cutoff, respectively (median +/- 2.5*MAD, colored in yellow). The readings beyond these cutoffs are considered as non-random stabilization or destabilization. A 30% change over baseline variance cutoff is set as the threshold (colored in green) for selecting significantly stabilized or destabilized hits. The responsive point for the hit protein is defined as the intersection between the ITDR_CETSA_ curve with the horizontal baseline, with the corresponding concentration value as the minimal dose threshold (MDT). (B) Levels (half-life) of spiked cAMP (1mM) in treated lysates over time. (C) Novel resolution of the cAMP-dependent PKA system’s interactions with cAMP and cGMP. cAMP-specific effects on PKA could be achieved and distinguished from cGMP by the relative differences in their MDT for most regulatory subunits (1) or through their differential effects on the biophysical stability of PRKARIIB (2). cGMP could destabilize RIIB by causing its separation from regulatory subunit binding protein (purple). Identification of SMAKA as a ligand for cGMP (3). The biophysical stabilization of SMAKA could be achieved through its phosphorylation (P) by PRKAC (C) upon cyclic nucleotide binding [35]. (D) Western blot (WB) ITDR_52_ of TYMS from lysate with dCMP versus dUMP with western blot. (E) T_m_ shift of TYMS induced by 2mM of dCMP, dTMP and dUMP. TSA: thermal stability assay. (F) Effect of dCMP versus dTMP on recombinant TYMS’s activity. (G) Binding site of dCMP and dUMP to TYMS. Crystal structure of TYMS complexed with dCMP, shown with 2Fo-Fc density map around dCMP contoured at 1.0 sigma. (H) Alphascreen ITDR_52_ of TYMS from lysate with dTMP, dUMP and dCMP with different incubation time. RLU: relative luminescence units, AU: arbitrary units.
(TIF)

S2 Fig Supporting data for NAD(P)(H) ITDRs. (A) Levels (half-life) of spiked NADPH (1mM) in treated lysates over time. (B) Domain alignment of NAD(P)(H) ITDR_CETSA_ hit proteins. Hit proteins that were detected in all 4 experiments were aligned according to their InterPro domains. The proteins formed 5 broad clusters (1–5): NAD(P) binding domain superfamily (1), FAD/NAD(P)-binding domain superfamily (2), NADP-dependent oxidoreductase domain superfamily (3), Aldehyde dehydrogenase domain (4), Protein-tyrosine phosphatase-like (5). Cluster 1 is split further into 5 subclusters: Short-chain dehydrogenase/reductase SDR (1A), no additional predominant domain (1B), GroES-like superfamily (1C), D-isomer specific 2-hydroxyacid dehydrogenase, NAD-binding domain (1D), 6-phosphogluconate dehydrogenase-like, C-terminal domain superfamily (1E). Hit ligands are indicated in red (stabilizing) or blue (destabilizing). Ligands in grey indicate potential protein-ligand interactions as per ITDR curves (S5 Plot) that did not meet the hit selection criteria. Non-hits are indicated with a dash (-). Annotated known hit proteins (underlined protein names), novel NAD(P)(H) hit (not underlined). (C) ITDR_CETSA_ curves of PTPases. PTPN1 (blue), PTPN11 (purple), PTPN12 (orange), ACP1 (green). (D) Subset of proteins used to determine the false negative rates for CETSA from the NADPH_ITDR52_, NADPH_ITDR58,_ control melt curves dataset experiments that are also NADPH annotated proteins. AU: arbitrary units.
(TIF)

S3 Fig Supporting data for dT-in-cell ITTRs. Effects of dT on the distribution of cells in different stages of cell cycle and protein expression versus abundance in cells treated with thymidine at 37°C. (A) Representative histograms showing intracellular DNA content of K562 cells after no treatment and 3h or 24h after treatment with 100mM thymidine respectively. Cells were treated with thymidine or control for 3h or 24h and then fixed and stained with propidium iodide and DNA content was analyzed using flow cytometry. Distribution of cells in different stages of the cell cycle after (B) 3h and (C) 24h. (D) The variability of protein expression has an anti-correlation with protein abundance in two biological replicates, suggesting that the observed expression variability could be attributable to technical variation because of low protein abundance. (E) Majority of the proteins did not show variation of protein expression greater than 10%. Thermal stability assay of purified recombinant full-length ABCF1 with (F) thymidine and its corresponding nucleotides or (G) adenine and its corresponding nucleotides.
(TIF)

S1 Plot Hit proteins for cAMP and/or cGMP as identified by ITDR_CETSA_. Only proteins that were found in both treatments are selected for hit list generation. Data is presented as two individual technical replicates for each condition from one representative experiment. Proteins highlighted in grey were not found in all cyclic nucleotide and deoxynucleotide datasets were omitted from the heatmap Fig 1B.
(PDF)

S2 Plot Top hit proteins affected by pH 6.5 and 8.5 as identified by CETSA shifts. Data is presented as two technical replicates (rep1 and rep2) per condition. While evaluating data collection and analysis schemes, we also noted a small common population of unanticipated hit proteins for some lysate experiments. We collected pH reference data sets and found that these lysate experiments had experienced small pH shifts in the aliquots with the highest compound concentrations, which might have led to changes in the biophysical stability of these proteins. We therefore included concentration cut-offs in the final analysis of these datasets, which attenuates the possible false effects in the hit generation.
(PDF)

S3 Plot Melting profiles of cyclic nucleotide and deoxynucleotide hit proteins at pH 6.5, pH 7.5 and pH 8.5. Plots are ordered according to complete replicates (page 1 and 2, rep1 and rep2) and incomplete replicates (page 3).
(PDF)

S4 Plot Hit proteins for the dNMPs and AMP as identified by ITDR_CETSA_. Only proteins that were found in both technical replicates are selected for hit list generation. Data is presented as the average of two technical replicates per condition from one representative experiment. Proteins highlighted in grey were not found in all cyclic nucleotide and deoxynucleotide datasets were omitted from the heatmap Fig 1B.
(PDF)

S5 Plot Hit proteins for the NAD(P)(H) as identified by ITDR_CETSA_. Only proteins that were found in both technical replicates are selected for hit list generation. Data is presented as the average of two technical replicates per condition from one representative experiment. Proteins highlighted in grey were not found in NAD(P)(H) datasets were omitted from the heatmap Fig 4B.
(PDF)

S6 Plot False negative estimate from ITDR_CETSA_ at 52°C and 58°C. We used the NADPH experiment to estimate the false negative rate in a given CETSA experiment, i.e. the number of proteins that bind to the said ligand but do not show a detectable change in thermal stability. We obtained a reviewed list of predicted human NADP binding protein based on the keyword annotation “NADP” from the protein database UniprotKB [67]. We also collected an ITDR data set at 58°C to get a better coverage of potential hits for proteins with high melting temperatures. This gave us a list of 38 proteins from the predicted NADPH binding protein list that were measured at both NADPH ITDR 52°C and 58°C datasets (S2C Fig and S8 Table), of which 11 did not come up in the list of hit proteins. One of these (ME1, P48163) gave a weak but significant response at both 52°C and 58°C and we consider this as a manually curated hit. Of the 10 remaining proteins, 8 proteins (NAXD, NAXE, PYCR3, CAT, GMPR, CRYZL1, KIAA191, NADK) had very high melting temperatures with no or < 25% melting at 58°C. These proteins would typically not be expected to respond in an ITDR at 58°C in spite of NADPH binding, but are potential responders at a higher temperature. For MICAL3 there is no direct binding data but the interaction of NADPH was reported with its close structural homolog MICAL. HSDL2 is a poorly characterised member of the short-chain dehydrogenase/reductase (SDR) subfamily. These two later proteins are candidates for non-responders in this data set. The above analysis suggests that the false negative rates for NADPH binding proteins might be very low, even below 10%. An analysis of the contribution of the 58°C experiment to hit rates showed a gain of 10 more novel hits, in addition of the 18 proteins identified in the 52°C ITDR hit list. For most of these proteins, the MDT for ITDR 52°C response was lower than at 58°C, up to 1.5 times. This is consistent with the temperature dependence of the CETSA response [25]. Interestingly, the fold changes compared to background were much greater in the ITDR 58°C dataset. This is due to the higher temperature where the background protein level was relatively lower.
(PDF)

S7 Plot Melting curves of proteins from the ITDR_CETSA_ at 52°C and 58°C false negative analysis subset (S2D Fig, S6 Plot, S8 and S9 Tables). (PDF)

S8 Plot Hit proteins as identified by in-cell ITDR_CETSA_ of thymidine-treated cells after 3 hours. Data is presented as two individual technical replicates of two biological replicates. In our preliminary experiments, we did see signs of shifts in the population of pH-sensitive proteins that were previously observed in the lysate experiments, indicating changes in intracellular pH. These pH-sensitive shifts were however not present in the subsequent more controlled experiments.
(PDF)

S1 Table Panel of protein-nucleotide interactions identified by ITDR_CETSA_. Nucleotides (green), nucleotides and cofactors NAD(P)(H) (red), cofactors NAD(P)(H) (blue). Relates to Figs 1–4.
(XLSX)

S2 Table List of hit proteins per ligand. Relates to Figs 1–4.
(XLSX)

S3 Table Melt curve parameters of cyclic nucleotide and deoxynucleotide hit proteins at pH 6.5 (pH6.1, pH6.2), pH 7.5 (pH7.1, pH7.2) and pH 8.5 (pH8.1, pH8.2). Relates to S2 and S3 Plots.
(XLSX)

S4 Table ITDR_CETSA_ parameters of cyclic nucleotide hit proteins. Relates to S1 Plot.
(XLSX)

S5 Table ITDR_CETSA_ parameters of deoxynucleotide hit proteins. Relates to S4 Plot.
(XLSX)

S6 Table Thermal shift assay (TSA) of purified proteins using differential static light scattering. Compound groups: nucleotides, deoxynucleosides, nucleosides, deoxynucleotides, nucleobases. Thermal shifts, ΔTagg, are given as changes in temperature. Values range from negative (blue) to positive (brown) ΔTagg. Relates to Figs 3 and 5.
(XLSX)

S7 Table ITDR_CETSA_ parameters of NAD(P)(H) hit proteins. Relates to Figs 4 and S2.
(XLSX)

S8 Table ITDR_CETSA_ parameters of NADPH_ITDR52_ and NADPH_ITDR58_ hit proteins. Relates to S2D Fig and S6 Plot.
(XLSX)

S9 Table Protein subset for false negative rate analysis in NADPH_ITDR52_ and NADPH_ITDR58_. Relates to S2D Fig and S6 Plot.
(XLSX)

S10 Table X-ray diffraction data and refinement statistics. *Highest resolution shell is shown in parenthesis. Relates to S1G Fig.
(XLSX)

S11 Table Ratio of change in protein expression at 37°C isotherm between treated and untreated conditions for proteins identified in the hit list for all nucleotides and NADPH. NA indicates that the protein was not detected and quantified for the corresponding metabolite treatment.
(XLSX)
