# Supplementary figures and images for: An efficient proteome-wide strategy for discovery and characterization of cellular nucleotide-protein interactions
Source: PLoS One. 2018 Dec 6;13(12):e0208273. doi: 10.1371/journal.pone.0208273 (PMC6283526; doi:10.1371/journal.pone.0208273)

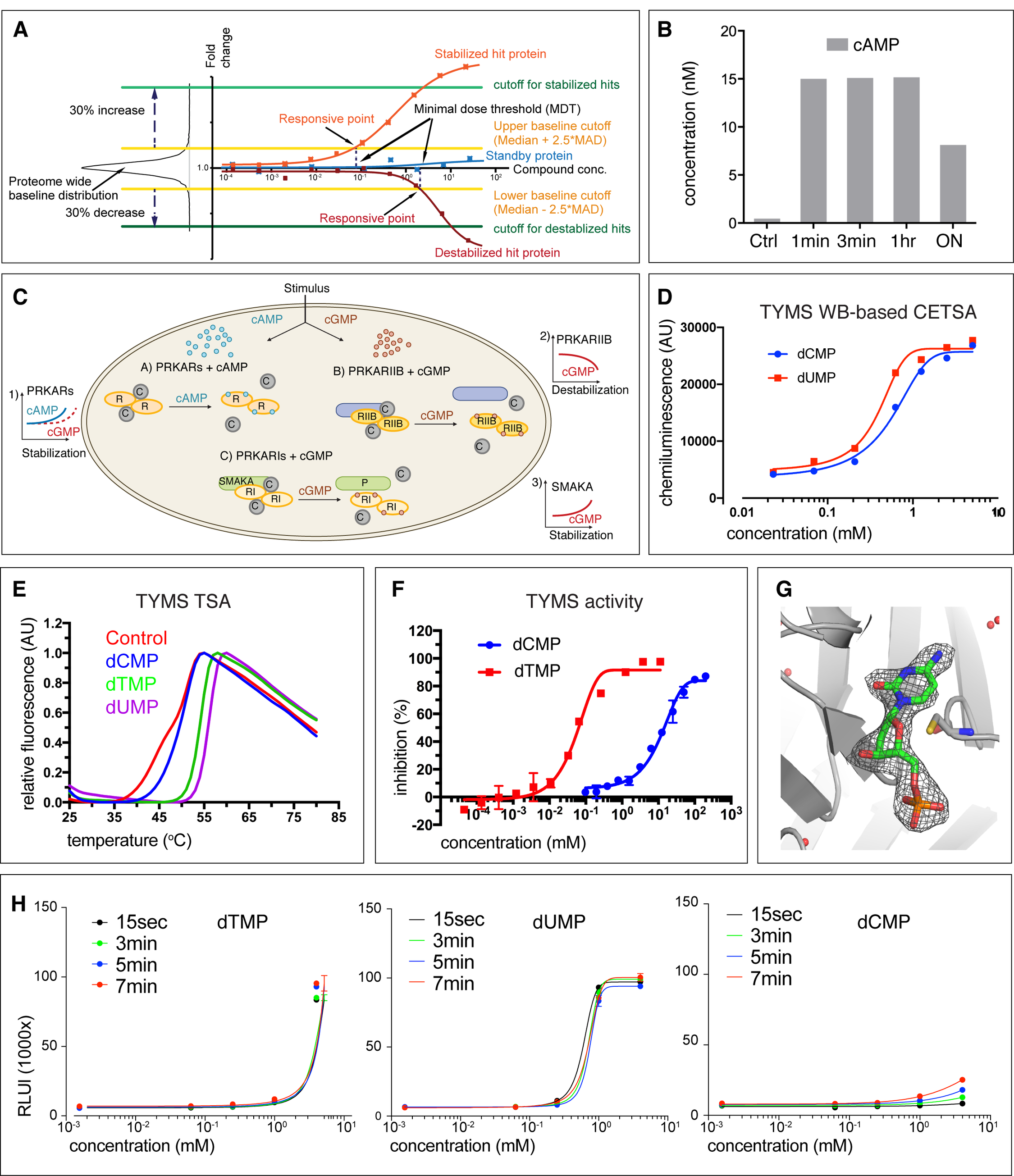

Supplement: S1 Fig — (A) Hit selection scheme in ITDRCETSA experiment. The readings from the lowest three concentration groups are used to derive an upper and lower baseline variance cutoff, respectively (median +/- 2.5*MAD, colored in yellow). The readings beyond these cutoffs are considered as non-random stabilization or destabilization. A 30% change over baseline variance cutoff is set as the threshold (colored in green) for selecting significantly stabilized or destabilized hits. The responsive point for the hit protein is defined as the intersection between the ITDRCETSA curve with the horizontal baseline, with the corresponding concentration value as the minimal dose threshold (MDT). (B) Levels (half-life) of spiked cAMP (1mM) in treated lysates over time. (C) Novel resolution of the cAMP-dependent PKA system’s interactions with cAMP and cGMP. cAMP-specific effects on PKA could be achieved and distinguished from cGMP by the relative differences in their MDT for most regulatory subunits (1) or through their differential effects on the biophysical stability of PRKARIIB (2). cGMP could destabilize RIIB by causing its separation from regulatory subunit binding protein (purple). Identification of SMAKA as a ligand for cGMP (3). The biophysical stabilization of SMAKA could be achieved through its phosphorylation (P) by PRKAC (C) upon cyclic nucleotide binding [35]. (D) Western blot (WB) ITDR52 of TYMS from lysate with dCMP versus dUMP with western blot. (E) Tm shift of TYMS induced by 2mM of dCMP, dTMP and dUMP. TSA: thermal stability assay. (F) Effect of dCMP versus dTMP on recombinant TYMS’s activity. (G) Binding site of dCMP and dUMP to TYMS. Crystal structure of TYMS complexed with dCMP, shown with 2Fo-Fc density map around dCMP contoured at 1.0 sigma. (H) Alphascreen ITDR52 of TYMS from lysate with dTMP, dUMP and dCMP with different incubation time. RLU: relative luminescence units, AU: arbitrary units. (TIF) [file pone.0208273.s001.tif]

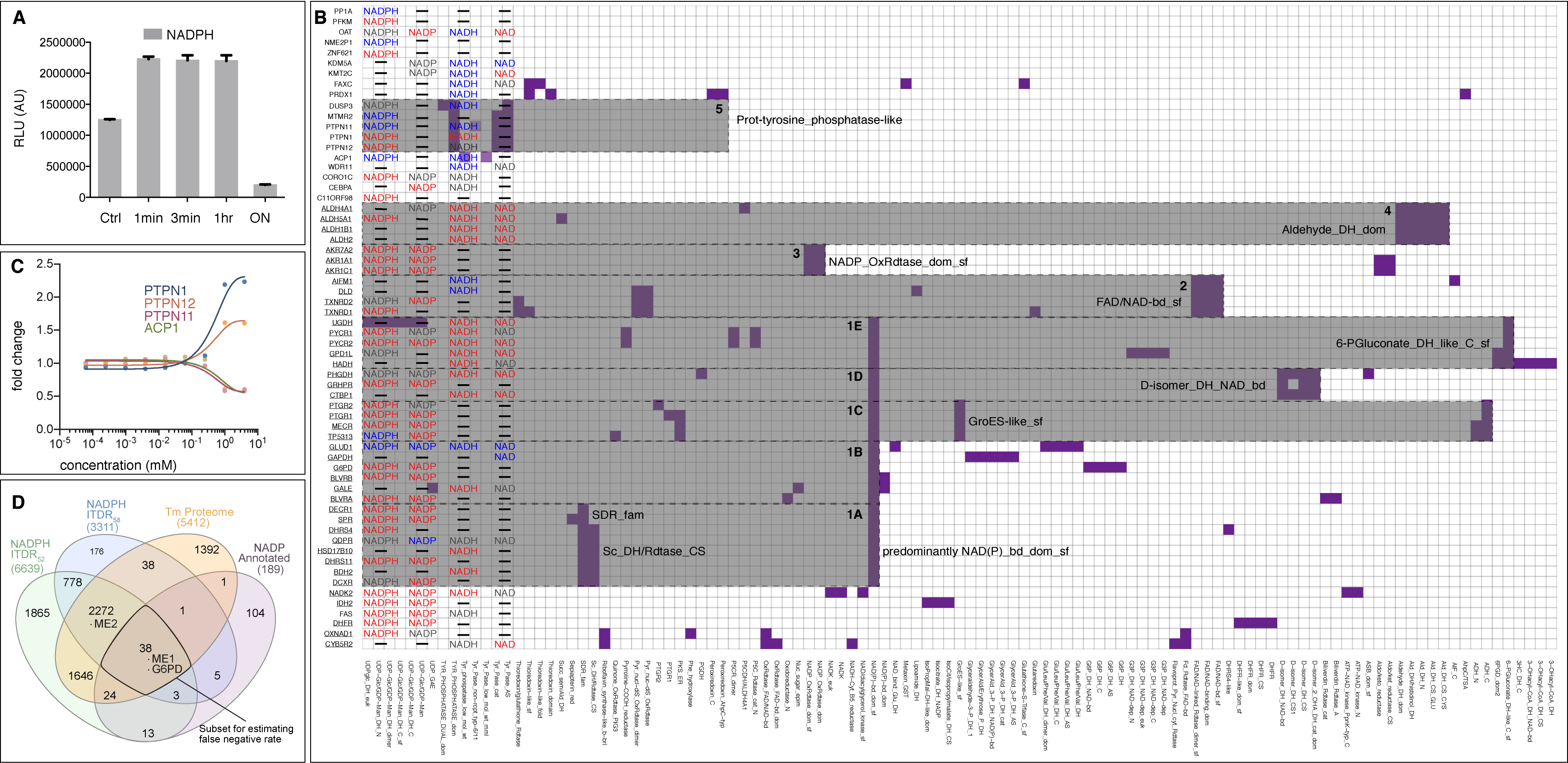

Supplement: S2 Fig — (A) Levels (half-life) of spiked NADPH (1mM) in treated lysates over time. (B) Domain alignment of NAD(P)(H) ITDRCETSA hit proteins. Hit proteins that were detected in all 4 experiments were aligned according to their InterPro domains. The proteins formed 5 broad clusters (1–5): NAD(P) binding domain superfamily (1), FAD/NAD(P)-binding domain superfamily (2), NADP-dependent oxidoreductase domain superfamily (3), Aldehyde dehydrogenase domain (4), Protein-tyrosine phosphatase-like (5). Cluster 1 is split further into 5 subclusters: Short-chain dehydrogenase/reductase SDR (1A), no additional predominant domain (1B), GroES-like superfamily (1C), D-isomer specific 2-hydroxyacid dehydrogenase, NAD-binding domain (1D), 6-phosphogluconate dehydrogenase-like, C-terminal domain superfamily (1E). Hit ligands are indicated in red (stabilizing) or blue (destabilizing). Ligands in grey indicate potential protein-ligand interactions as per ITDR curves (S5 Plot) that did not meet the hit selection criteria. Non-hits are indicated with a dash (-). Annotated known hit proteins (underlined protein names), novel NAD(P)(H) hit (not underlined). (C) ITDRCETSA curves of PTPases. PTPN1 (blue), PTPN11 (purple), PTPN12 (orange), ACP1 (green). (D) Subset of proteins used to determine the false negative rates for CETSA from the NADPHITDR52, NADPHITDR58, control melt curves dataset experiments that are also NADPH annotated proteins. AU: arbitrary units. (TIF) [file pone.0208273.s002.tif]

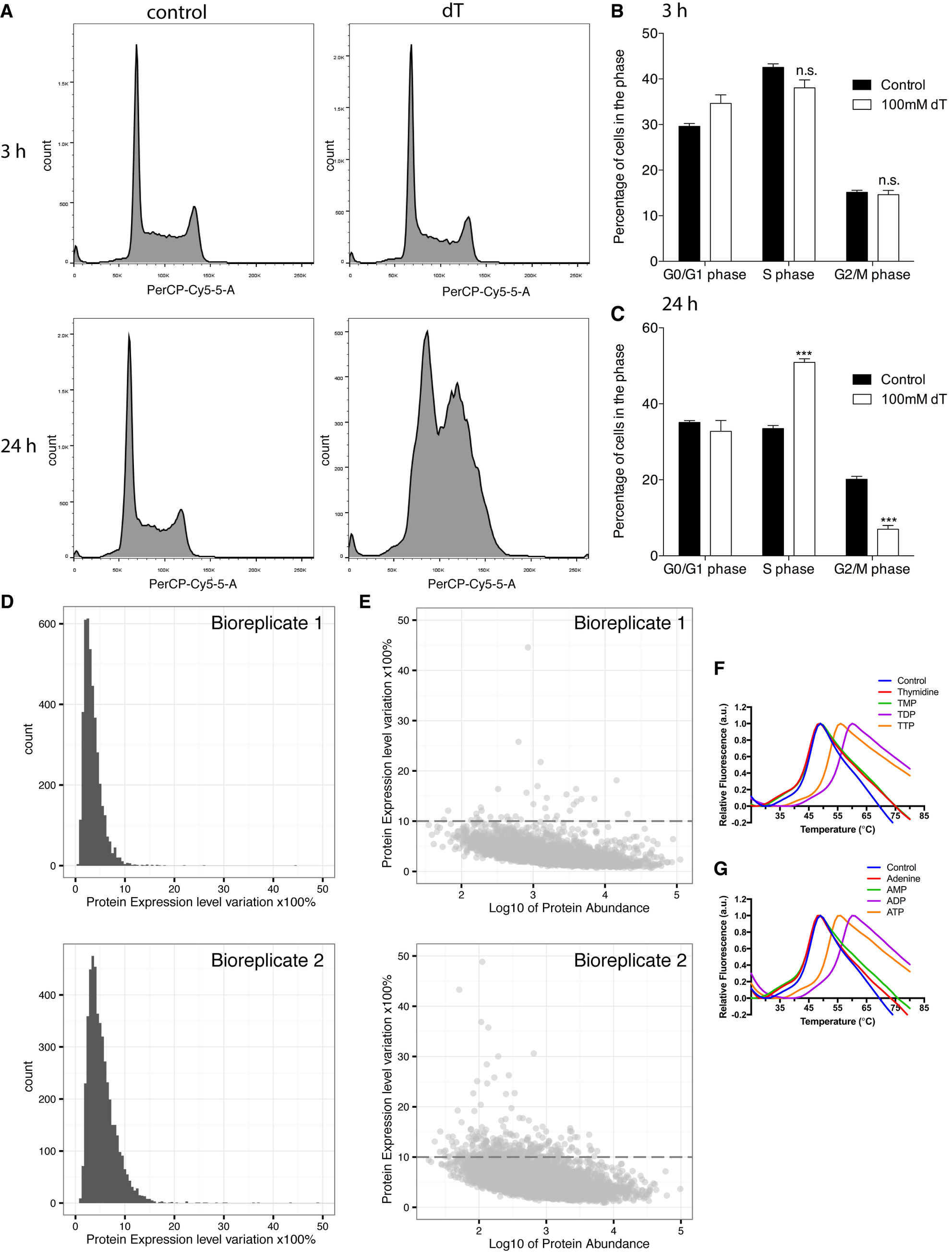

Supplement: S3 Fig — Effects of dT on the distribution of cells in different stages of cell cycle and protein expression versus abundance in cells treated with thymidine at 37°C. (A) Representative histograms showing intracellular DNA content of K562 cells after no treatment and 3h or 24h after treatment with 100mM thymidine respectively. Cells were treated with thymidine or control for 3h or 24h and then fixed and stained with propidium iodide and DNA content was analyzed using flow cytometry. Distribution of cells in different stages of the cell cycle after (B) 3h and (C) 24h. (D) The variability of protein expression has an anti-correlation with protein abundance in two biological replicates, suggesting that the observed expression variability could be attributable to technical variation because of low protein abundance. (E) Majority of the proteins did not show variation of protein expression greater than 10%. Thermal stability assay of purified recombinant full-length ABCF1 with (F) thymidine and its corresponding nucleotides or (G) adenine and its corresponding nucleotides. (TIF) [file pone.0208273.s003.tif]

ITDR CETSA data plotting

Non-denatured protein fraction

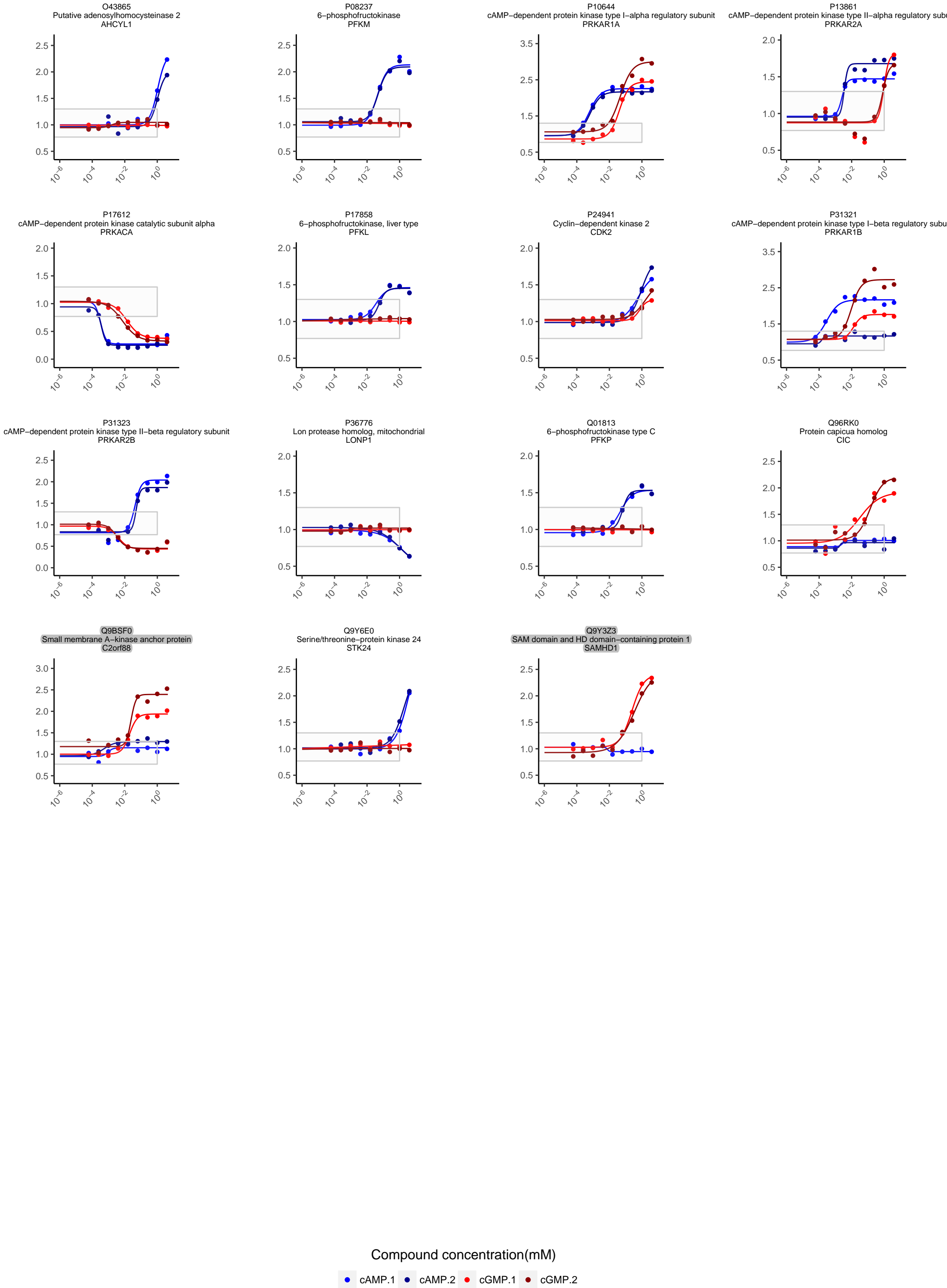

Supplement: S1 Plot — Only proteins that were found in both treatments are selected for hit list generation. Data is presented as two individual technical replicates for each condition from one representative experiment. Proteins highlighted in grey were not found in all cyclic nucleotide and deoxynucleotide datasets were omitted from the heatmap Fig 1B. (PDF) [file pone.0208273.s004.pdf]
