# Supplementary material for: An efficient proteome-wide strategy for discovery and characterization of cellular nucleotide-protein interactions
Source: PLoS One. 2018 Dec 6;13(12):e0208273. doi: 10.1371/journal.pone.0208273 (PMC6283526; doi:10.1371/journal.pone.0208273)
Supplement: S2 Plot — Data is presented as two technical replicates (rep1 and rep2) per condition. While evaluating data collection and analysis schemes, we also noted a small common population of unanticipated hit proteins for some lysate experiments. We collected pH reference data sets and found that these lysate experiments had experienced small pH shifts in the aliquots with the highest compound concentrations, which might have led to changes in the biophysical stability of these proteins. We therefore included concentration cut-offs in the final analysis of these datasets, which attenuates the possible false effects in the hit generation. (PDF) [file pone.0208273.s005.pdf]

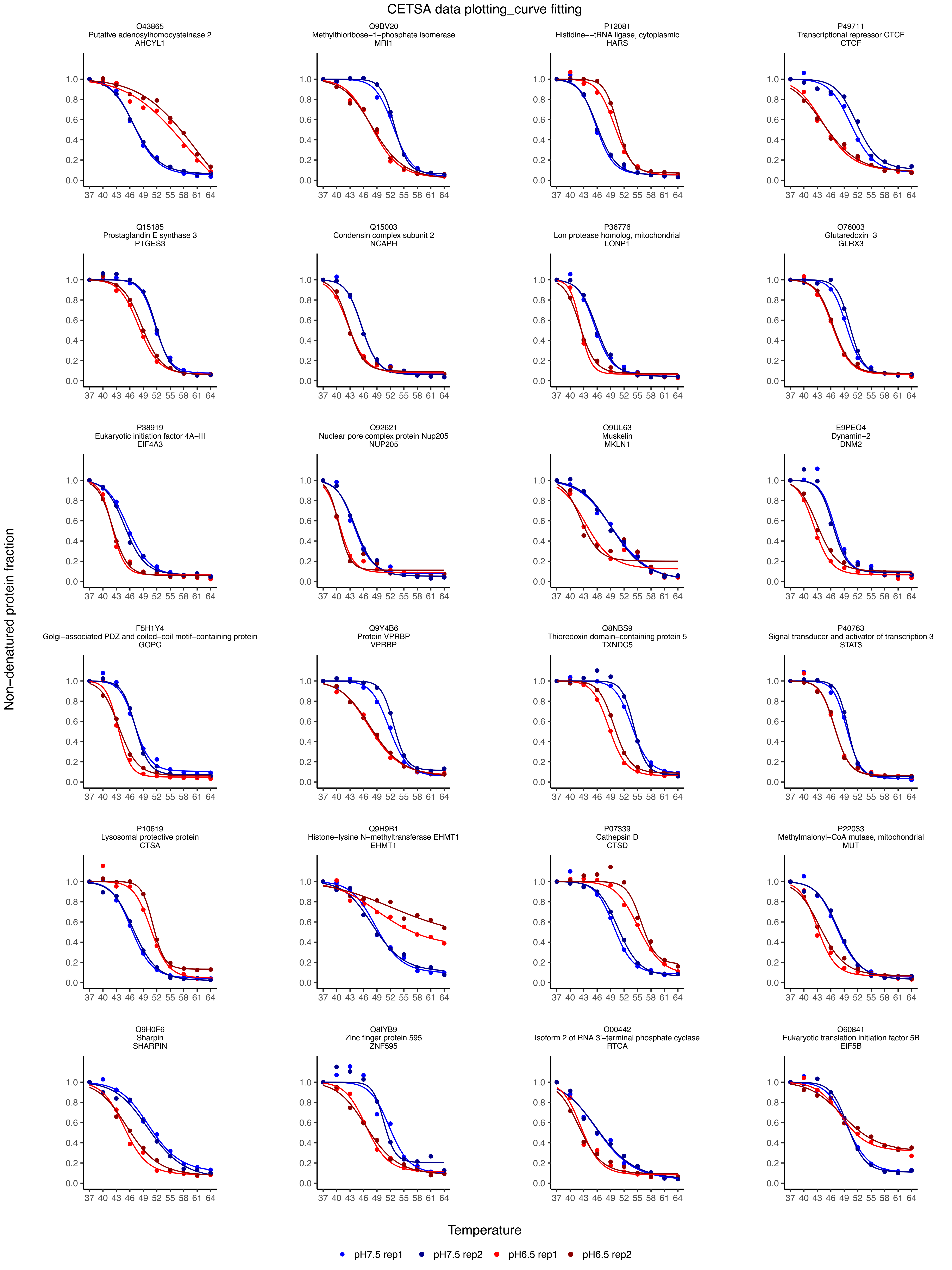
CETSA data plotting_curve fitting
O43865
Putative adenosylhomocysteinase 2
AHCYL1
Q9BV20
Methylthioribose−1−phosphate isomerase
MRI1
P12081
Histidine−−tRNA ligase, cytoplasmic
HARS
P49711
Transcriptional repressor CTCF
CTCF
Q15185
Prostaglandin E synthase 3
PTGES3
Q15003
Condensin complex subunit 2
NCAPH
P36776
Lon protease homolog, mitochondrial
LONP1
O76003
Glutaredoxin−3
GLRX3
P38919
Eukaryotic initiation factor 4A−III
EIF4A3
Q92621
Nuclear pore complex protein Nup205
NUP205
Q9UL63
Muskelin
MKLN1
E9PEQ4
Dynamin−2
DNM2
F5H1Y4
Golgi−associated PDZ and coiled−coil motif−containing protein
GOPC
Q9Y4B6
Protein VPRBP
VPRBP
Q8NBS9
Thioredoxin domain−containing protein 5
TXNDC5
P40763
Signal transducer and activator of transcription 3
STAT3
P10619
Lysosomal protective protein
CTSA
Q9H9B1
Histone−lysine N−methyltransferase EHMT1
EHMT1
P07339
Cathepsin D
CTSD
P22033
Methylmalonyl−CoA mutase, mitochondrial
MUT
Q9H0F6
Sharpin
SHARPIN
Q8IYB9
Zinc finger protein 595
ZNF595
O00442
Isoform 2 of RNA 3'−terminal phosphate cyclase
RTCA
O60841
Eukaryotic translation initiation factor 5B
EIF5B
0.0 0.2 0.4 0.6 0.8 1.0
37 40 43 46 49 52 55 58 61 64
Non−denatured protein fraction
Temperature
pH7.5 rep1
pH7.5 rep2
pH6.5 rep1
pH6.5 rep2

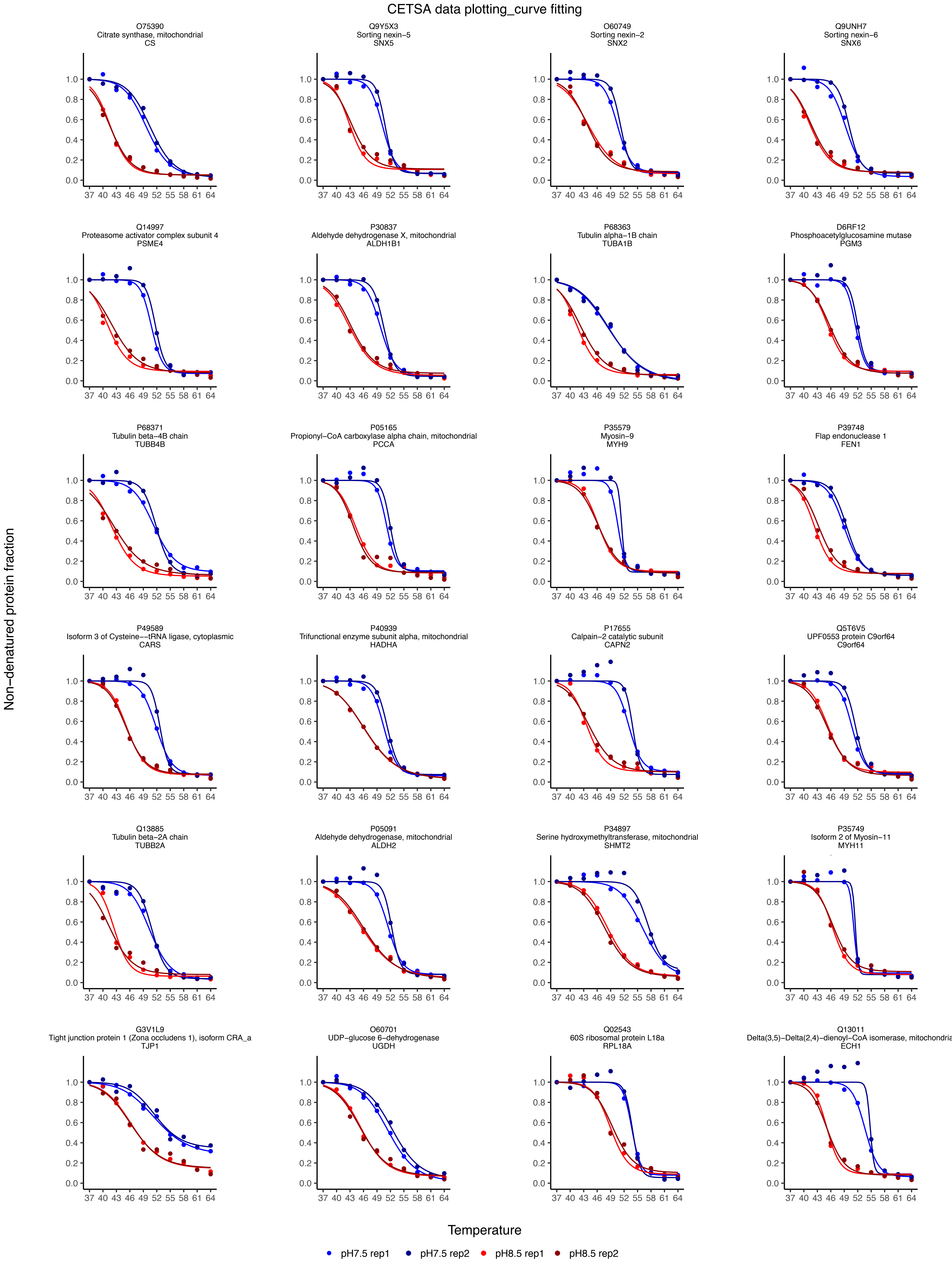
CETSA data plotting_curve fitting
O75390
Citrate synthase, mitochondrial
CS
Q9Y5X3
Sorting nexin−5
SNX5
O60749
Sorting nexin−2
SNX2
Q9UNH7
Sorting nexin−6
SNX6
Q14997
Proteasome activator complex subunit 4
PSME4
P30837
Aldehyde dehydrogenase X, mitochondrial
ALDH1B1
P68363
Tubulin alpha−1B chain
TUBA1B
D6RF12
Phosphoacetylglucosamine mutase
PGM3
P68371
Tubulin beta−4B chain
TUBB4B
P05165
Propionyl−CoA carboxylase alpha chain, mitochondrial
PCCA
P35579
Myosin−9
MYH9
P39748
Flap endonuclease 1
FEN1
P49589
Isoform 3 of Cysteine−−tRNA ligase, cytoplasmic
CARS
P40939
Trifunctional enzyme subunit alpha, mitochondrial
HADHA
P17655
Calpain−2 catalytic subunit
CAPN2
Q5T6V5
UPF0553 protein C9orf64
C9orf64
Q13885
Tubulin beta−2A chain
TUBB2A
P05091
Aldehyde dehydrogenase, mitochondrial
ALDH2
P34897
Serine hydroxymethyltransferase, mitochondrial
SHMT2
P35749
Isoform 2 of Myosin−11
MYH11
G3V1L9
Tight junction protein 1 (Zona occludens 1), isoform CRA_a
TJP1
O60701
UDP−glucose 6−dehydrogenase
UGDH
Q02543
60S ribosomal protein L18a
RPL18A
Q13011
Delta(3,5)−Delta(2,4)−dienoyl−CoA isomerase, mitochondria
ECH1
Non−denatured protein fraction
Temperature
pH7.5 rep1
pH7.5 rep2
pH8.5 rep1
pH8.5 rep2
