# Supplementary material for: An efficient proteome-wide strategy for discovery and characterization of cellular nucleotide-protein interactions
Source: PLoS One. 2018 Dec 6;13(12):e0208273. doi: 10.1371/journal.pone.0208273 (PMC6283526; doi:10.1371/journal.pone.0208273)
Supplement: S3 Plot — Plots are ordered according to complete replicates (page 1 and 2, rep1 and rep2) and incomplete replicates (page 3). (PDF) [file pone.0208273.s006.pdf]

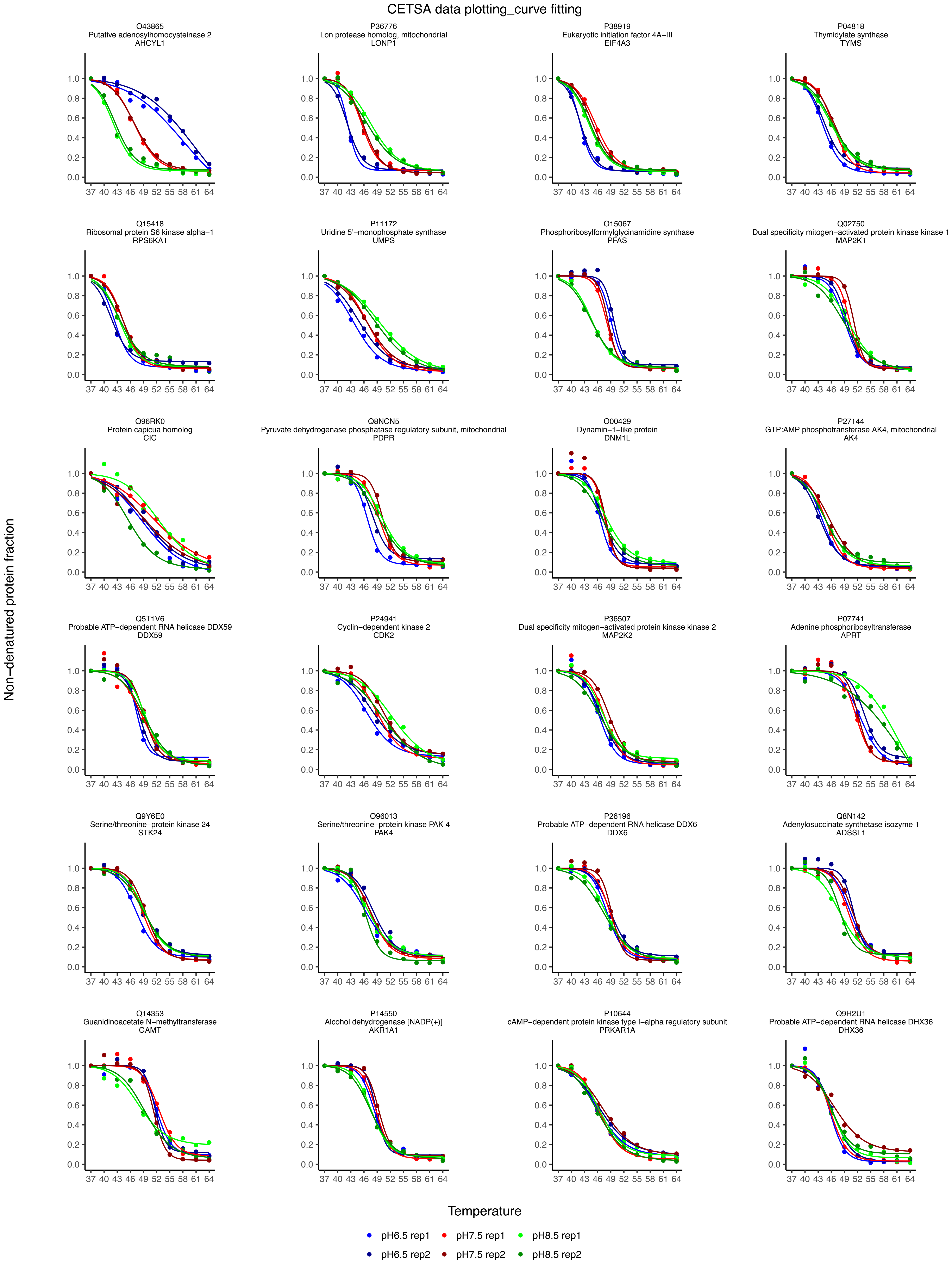
CETSA data plotting_curve fitting
O43865
Putative adenosylhomocysteinase 2
AHCYL1
P36776
Lon protease homolog, mitochondrial
LONP1
P38919
Eukaryotic initiation factor 4A−III
EIF4A3
P04818
Thymidylate synthase
TYMS
Q15418
Ribosomal protein S6 kinase alpha−1
RPS6KA1
P11172
Uridine 5'−monophosphate synthase
UMPS
O15067
Phosphoribosylformylglycinamidine synthase
PFAS
Q02750
Dual specificity mitogen−activated protein kinase kinase 1
MAP2K1
Q96RK0
Protein capicua homolog
CIC
Q8NCN5
Pyruvate dehydrogenase phosphatase regulatory subunit, mitochondrial
PDPR
O00429
Dynamin−1−like protein
DNM1L
P27144
GTP:AMP phosphotransferase AK4, mitochondrial
AK4
Q5T1V6
Probable ATP−dependent RNA helicase DDX59
DDX59
P24941
Cyclin−dependent kinase 2
CDK2
P36507
Dual specificity mitogen−activated protein kinase kinase 2
MAP2K2
P07741
Adenine phosphoribosyltransferase
APRT
Q9Y6E0
Serine/threonine−protein kinase 24
STK24
O96013
Serine/threonine−protein kinase PAK 4
PAK4
P26196
Probable ATP−dependent RNA helicase DDX6
DDX6
Q8N142
Adenylosuccinate synthetase isozyme 1
ADSSL1
Q14353
Guanidinoacetate N−methyltransferase
GAMT
P14550
Alcohol dehydrogenase [NADP(+)]
AKR1A1
P10644
cAMP−dependent protein kinase type I−alpha regulatory subunit
PRKAR1A
Q9H2U1
Probable ATP−dependent RNA helicase DHX36
DHX36
0.0 0.2 0.4 0.6 0.8 1.0
37 40 43 46 49 52 55 58 61 64
Non−denatured protein fraction
Temperature
pH6.5 rep1
pH7.5 rep1
pH8.5 rep1
pH6.5 rep2
pH7.5 rep2
pH8.5 rep2

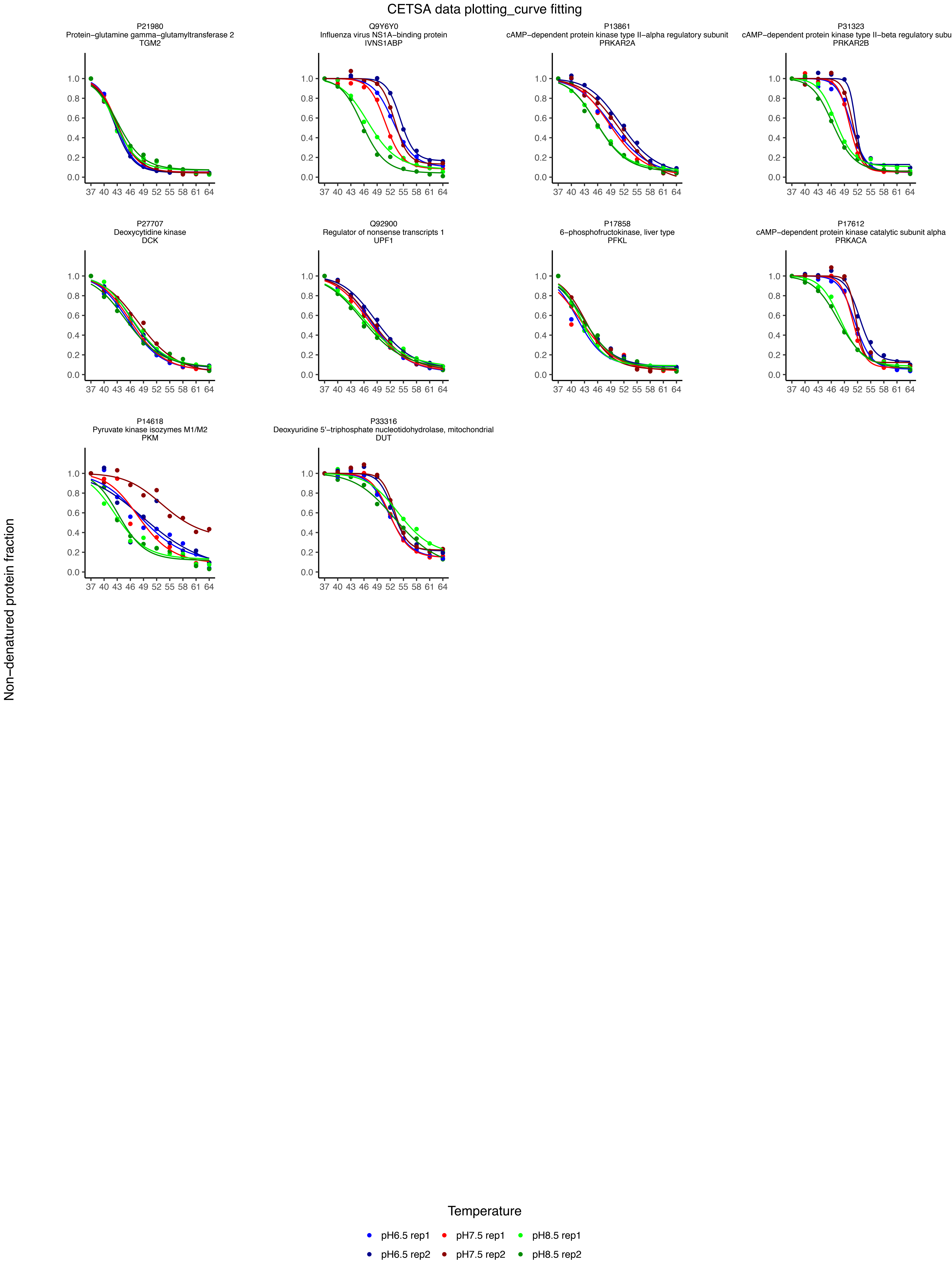
CETSA data plotting_curve fitting
P21980
Protein−glutamine gamma−glutamyltransferase 2
TGM2
Q9Y6Y0
Influenza virus NS1A−binding protein
IVNS1ABP
P13861
cAMP−dependent protein kinase type II−alpha regulatory subunit
PRKAR2A
P31323
cAMP−dependent protein kinase type II−beta regulatory subu
PRKAR2B
P27707
Deoxycytidine kinase
DCK
Q92900
Regulator of nonsense transcripts 1
UPF1
P17858
6−phosphofructokinase, liver type
PFKL
P17612
cAMP−dependent protein kinase catalytic subunit alpha
PRKACA
P14618
Pyruvate kinase isozymes M1/M2
PKM
P33316
Deoxyuridine 5'−triphosphate nucleotidohydrolase, mitochondrial
DUT
0.0
0.2
0.4
0.6
0.8
1.0
37 40 43 46 49 52 55 58 61 64
Non−denatured protein fraction
Temperature
pH6.5 rep1
pH7.5 rep1
pH8.5 rep1
pH6.5 rep2
pH7.5 rep2
pH8.5 rep2

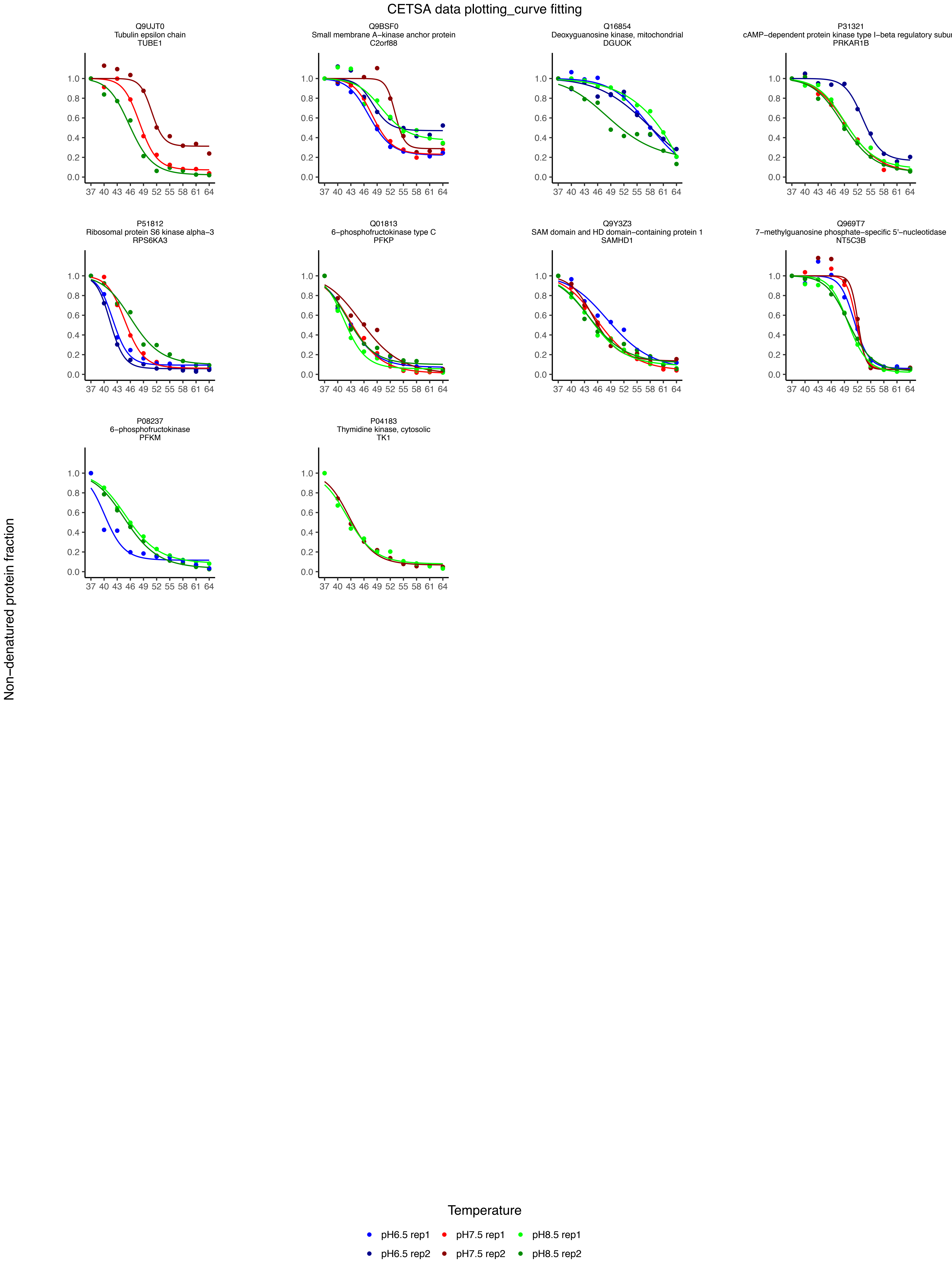
CETSA data plotting_curve fitting
Q9UJT0
Tubulin epsilon chain
TUBE1
Q9BSF0
Small membrane A−kinase anchor protein
C2orf88
Q16854
Deoxyguanosine kinase, mitochondrial
DGUOK
P31321
cAMP−dependent protein kinase type I−beta regulatory subu
PRKAR1B
P51812
Ribosomal protein S6 kinase alpha−3
RPS6KA3
Q01813
6−phosphofructokinase type C
PFKP
Q9Y3Z3
SAM domain and HD domain−containing protein 1
SAMHD1
Q969T7
7−methylguanosine phosphate−specific 5'−nucleotidase
NT5C3B
P08237
6−phosphofructokinase
PFKM
P04183
Thymidine kinase, cytosolic
TK1
0.0 0.2 0.4 0.6 0.8 1.0
37 40 43 46 49 52 55 58 61 64
Non−denatured protein fraction
Temperature
pH6.5 rep1
pH7.5 rep1
pH8.5 rep1
pH6.5 rep2
pH7.5 rep2
pH8.5 rep2
