# Supplementary material for: An efficient proteome-wide strategy for discovery and characterization of cellular nucleotide-protein interactions
Source: PLoS One. 2018 Dec 6;13(12):e0208273. doi: 10.1371/journal.pone.0208273 (PMC6283526; doi:10.1371/journal.pone.0208273)
Supplement: S4 Plot — Only proteins that were found in both technical replicates are selected for hit list generation. Data is presented as the average of two technical replicates per condition from one representative experiment. Proteins highlighted in grey were not found in all cyclic nucleotide and deoxynucleotide datasets were omitted from the heatmap Fig 1B. (PDF) [file pone.0208273.s007.pdf]

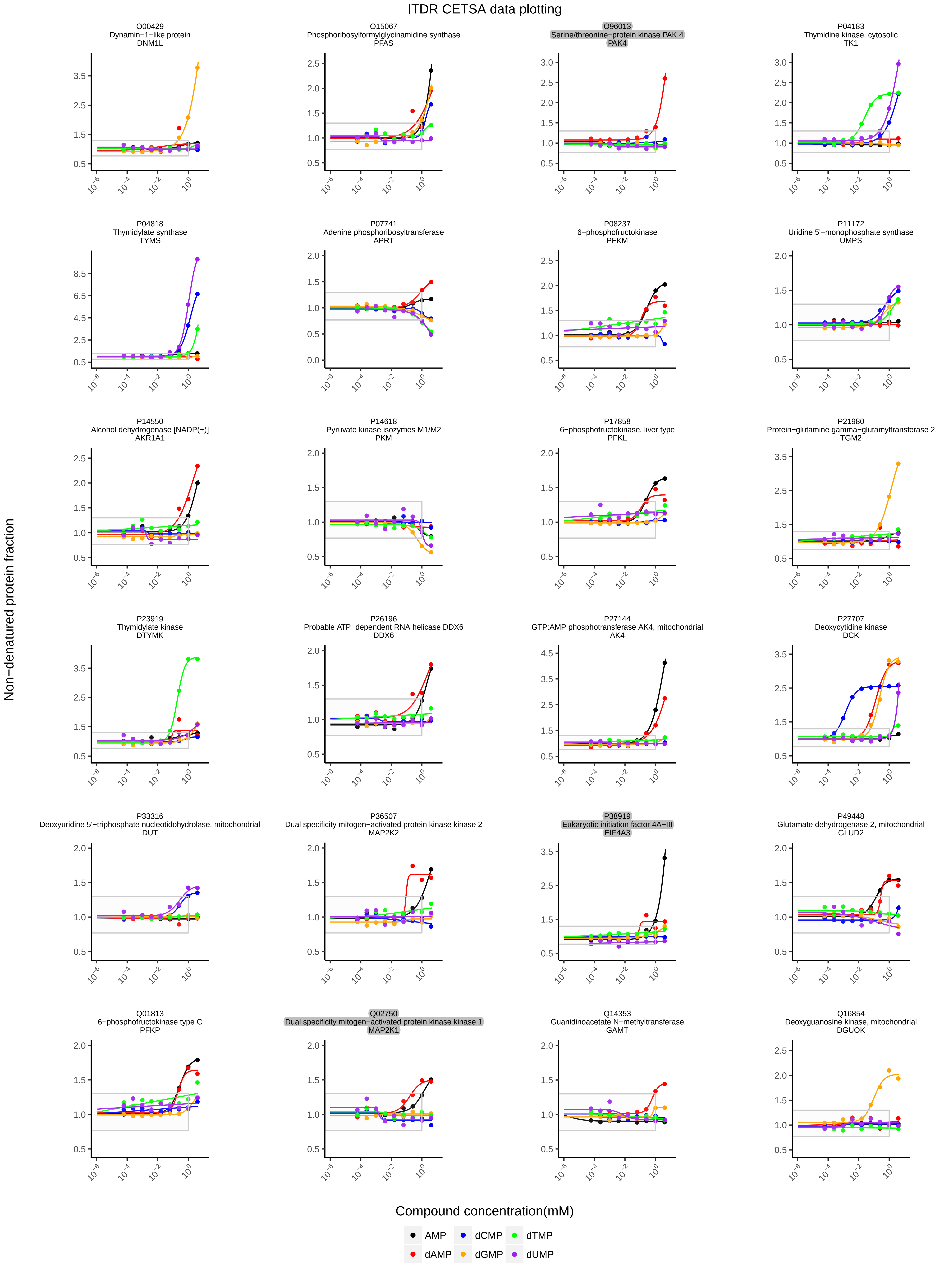
ITDR CETSA data plotting
O00429
Dynamin−1−like protein
DNM1L
O15067
Phosphoribosylformylglycinamidine synthase
PFAS
O96013
Serine/threonine−protein kinase PAK 4
PAK4
P04183
Thymidine kinase, cytosolic
TK1
P04818
Thymidylate synthase
TYMS
P07741
Adenine phosphoribosyltransferase
APRT
P08237
6−phosphofructokinase
PFKM
P11172
Uridine 5'−monophosphate synthase
UMPS
P14550
Alcohol dehydrogenase [NADP(+)]
AKR1A1
P14618
Pyruvate kinase isozymes M1/M2
PKM
P17858
6−phosphofructokinase, liver type
PFKL
P21980
Protein−glutamine gamma−glutamyltransferase 2
TGM2
P23919
Thymidylate kinase
DTYMK
P26196
Probable ATP−dependent RNA helicase DDX6
DDX6
P27144
GTP:AMP phosphotransferase AK4, mitochondrial
AK4
P27707
Deoxycytidine kinase
DCK
P33316
Deoxyuridine 5'−triphosphate nucleotidohydrolase, mitochondrial
DUT
P36507
Dual specificity mitogen−activated protein kinase kinase 2
MAP2K2
P38919
Eukaryotic initiation factor 4A−III
EIF4A3
P49448
Glutamate dehydrogenase 2, mitochondrial
GLUD2
Q01813
6−phosphofructokinase type C
PFKP
Q02750
Dual specificity mitogen−activated protein kinase kinase 1
MAP2K1
Q14353
Guanidinoacetate N−methyltransferase
GAMT
Q16854
Deoxyguanosine kinase, mitochondrial
DGUOK
Non−denatured protein fraction
Compound concentration(mM)
AMP
dCMP
dTMP
dAMP
dGMP
dUMP

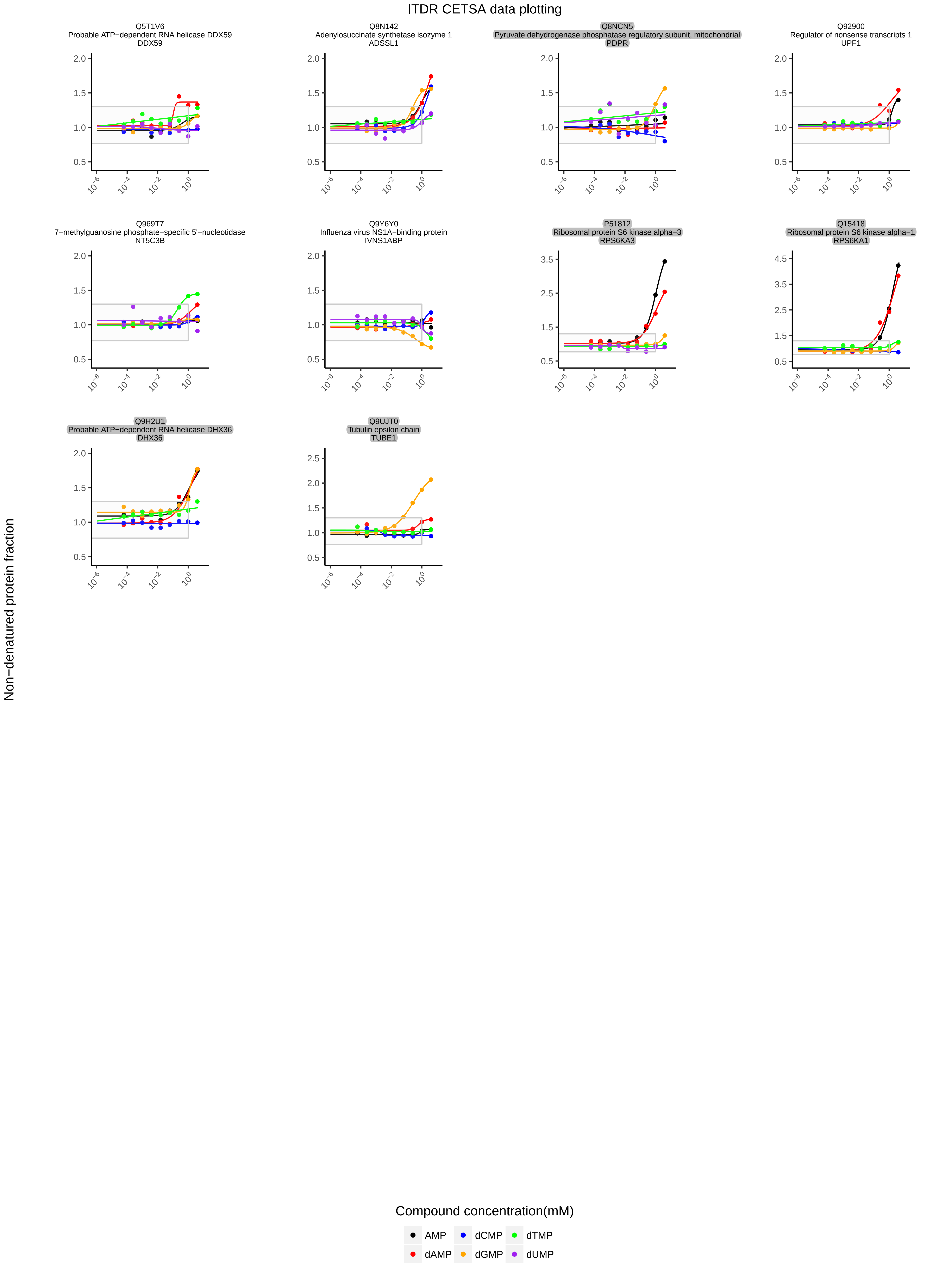
ITDR CETSA data plotting
Q5T1V6
Probable ATP−dependent RNA helicase DDX59
DDX59
Q8N142
Adenylosuccinate synthetase isozyme 1
ADSSL1
Q8NCN5
Pyruvate dehydrogenase phosphatase regulatory subunit, mitochondrial
PDPR
Q92900
Regulator of nonsense transcripts 1
UPF1
Q969T7
7−methylguanosine phosphate−specific 5'−nucleotidase
NT5C3B
Q9Y6Y0
Influenza virus NS1A−binding protein
IVNS1ABP
P51812
Ribosomal protein S6 kinase alpha−3
RPS6KA3
Q15418
Ribosomal protein S6 kinase alpha−1
RPS6KA1
Q9H2U1
Probable ATP−dependent RNA helicase DHX36
DHX36
Q9UJT0
Tubulin epsilon chain
TUBE1
Non−denatured protein fraction
Compound concentration(mM)
AMP
dCMP
dTMP
dAMP
dGMP
dUMP
