# Supplementary material for: An efficient proteome-wide strategy for discovery and characterization of cellular nucleotide-protein interactions
Source: PLoS One. 2018 Dec 6;13(12):e0208273. doi: 10.1371/journal.pone.0208273 (PMC6283526; doi:10.1371/journal.pone.0208273)
Supplement: S5 Plot — Only proteins that were found in both technical replicates are selected for hit list generation. Data is presented as the average of two technical replicates per condition from one representative experiment. Proteins highlighted in grey were not found in NAD(P)(H) datasets were omitted from the heatmap Fig 4B. (PDF) [file pone.0208273.s008.pdf]

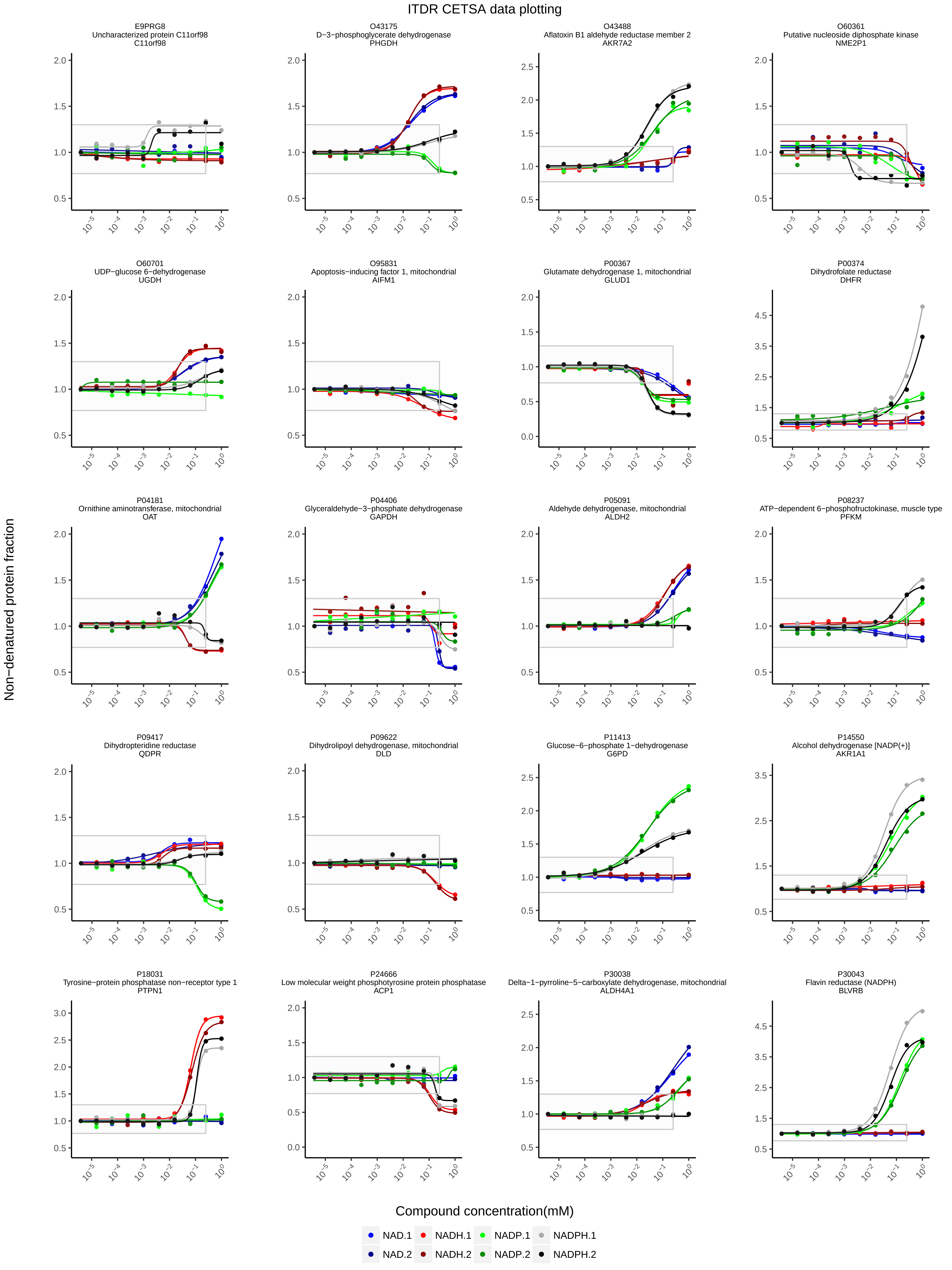
ITDR CETSA data plotting
E9PRG8
Uncharacterized protein C11orf98
C11orf98
O43175
D−3−phosphoglycerate dehydrogenase
PHGDH
O43488
Aflatoxin B1 aldehyde reductase member 2
AKR7A2
O60361
Putative nucleoside diphosphate kinase
NME2P1
O60701
UDP−glucose 6−dehydrogenase
UGDH
O95831
Apoptosis−inducing factor 1, mitochondrial
AIFM1
P00367
Glutamate dehydrogenase 1, mitochondrial
GLUD1
P00374
Dihydrofolate reductase
DHFR
P04181
Ornithine aminotransferase, mitochondrial
OAT
P04406
Glyceraldehyde−3−phosphate dehydrogenase
GAPDH
P05091
Aldehyde dehydrogenase, mitochondrial
ALDH2
P08237
ATP−dependent 6−phosphofructokinase, muscle type
PFKM
P09417
Dihydropteridine reductase
QDPR
P09622
Dihydrolipoyl dehydrogenase, mitochondrial
DLD
P11413
Glucose−6−phosphate 1−dehydrogenase
G6PD
P14550
Alcohol dehydrogenase [NADP(+)]
AKR1A1
P18031
Tyrosine−protein phosphatase non−receptor type 1
PTPN1
P24666
Low molecular weight phosphotyrosine protein phosphatase
ACP1
P30038
Delta−1−pyrroline−5−carboxylate dehydrogenase, mitochondrial
ALDH4A1
P30043
Flavin reductase (NADPH)
BLVRB
Non−denatured protein fraction
Compound concentration(mM)
NAD.1
NADH.1
NADP.1
NADPH.1
NAD.2
NADH.2
NADP.2
NADPH.2

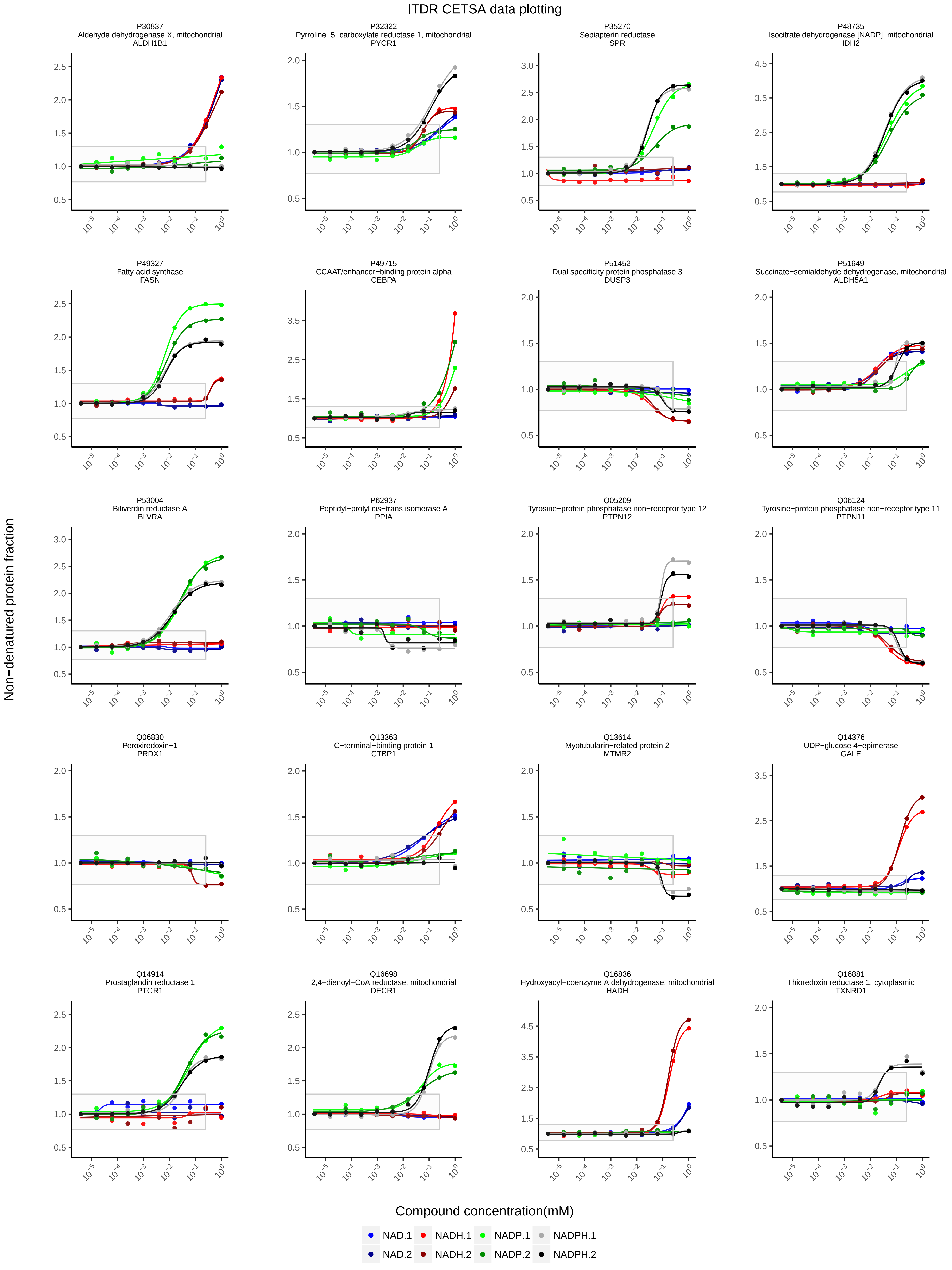
ITDR CETSA data plotting
P30837
Aldehyde dehydrogenase X, mitochondrial
ALDH1B1
P32322
Pyrroline−5−carboxylate reductase 1, mitochondrial
PYCR1
P35270
Sepiapterin reductase
SPR
P48735
Isocitrate dehydrogenase [NADP], mitochondrial
IDH2
P49327
Fatty acid synthase
FASN
P49715
CCAAT/enhancer−binding protein alpha
CEBPA
P51452
Dual specificity protein phosphatase 3
DUSP3
P51649
Succinate−semialdehyde dehydrogenase, mitochondrial
ALDH5A1
P53004
Biliverdin reductase A
BLVRA
P62937
Peptidyl−prolyl cis−trans isomerase A
PPIA
Q05209
Tyrosine−protein phosphatase non−receptor type 12
PTPN12
Q06124
Tyrosine−protein phosphatase non−receptor type 11
PTPN11
Q06830
Peroxiredoxin−1
PRDX1
Q13363
C−terminal−binding protein 1
CTBP1
Q13614
Myotubularin−related protein 2
MTMR2
Q14376
UDP−glucose 4−epimerase
GALE
Q14914
Prostaglandin reductase 1
PTGR1
Q16698
2,4−dienoyl−CoA reductase, mitochondrial
DECR1
Q16836
Hydroxyacyl−coenzyme A dehydrogenase, mitochondrial
HADH
Q16881
Thioredoxin reductase 1, cytoplasmic
TXNRD1
Non−denatured protein fraction
Compound concentration(mM)
NAD.1
NADH.1
NADP.1
NADPH.1
NAD.2
NADH.2
NADP.2
NADPH.2

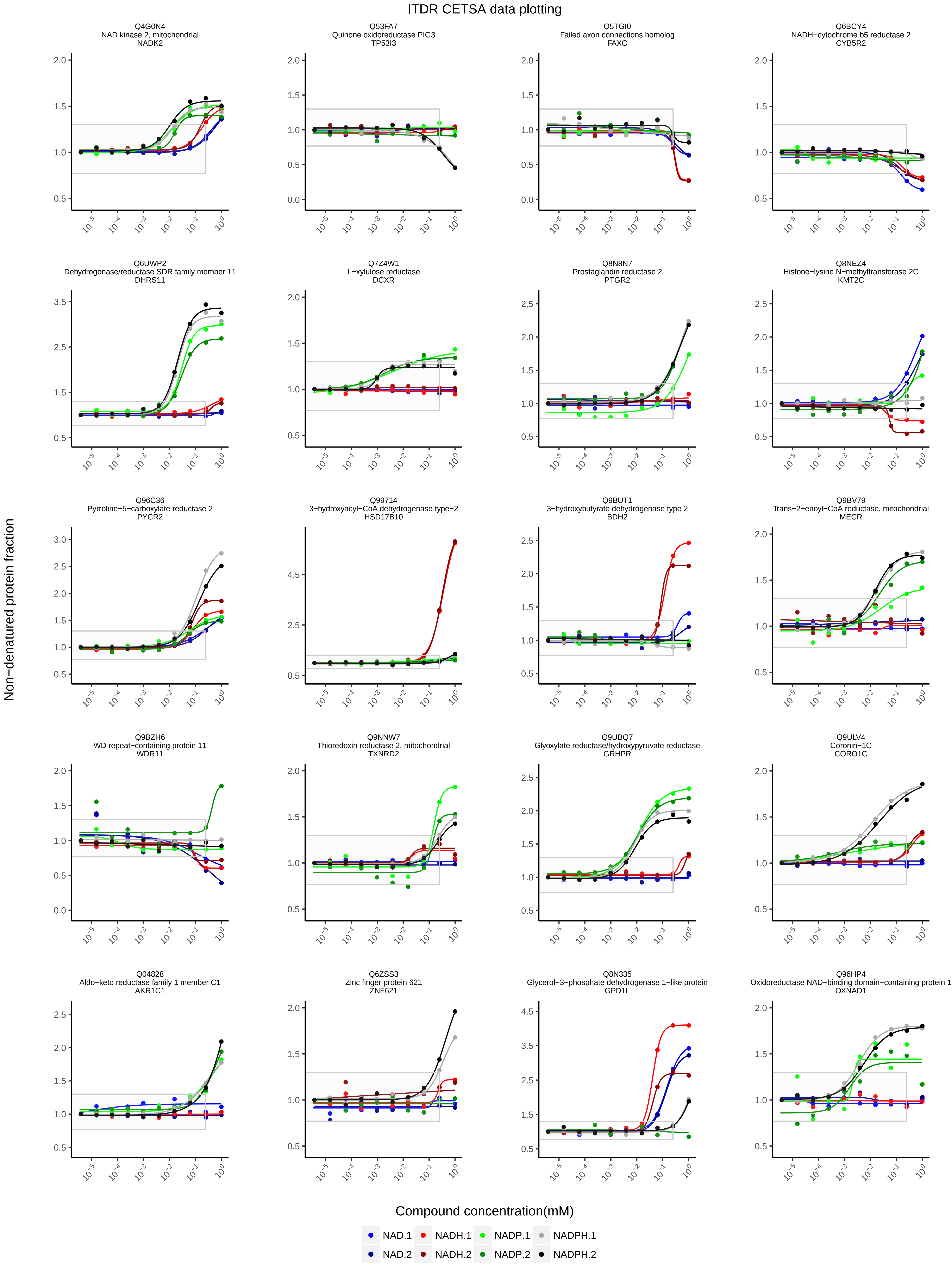

ITDR CETSA data plotting
Q4G0N4
NAD kinase 2, mitochondrial
NADK2
Q53FA7
Quinone oxidoreductase PIG3
TP53I3
Q5TGI0
Failed axon connections homolog
FAXC
Q6BCY4
NADH−cytochrome b5 reductase 2
CYB5R2
Q6UWP2
Dehydrogenase/reductase SDR family member 11
DHRS11
Q7Z4W1
L−xylulose reductase
DCXR
Q8N8N7
Prostaglandin reductase 2
PTGR2
Q8NEZ4
Histone−lysine N−methyltransferase 2C
KMT2C
Q96C36
Pyrroline−5−carboxylate reductase 2
PYCR2
Q99714
3−hydroxyacyl−CoA dehydrogenase type−2
HSD17B10
Q9BUT1
3−hydroxybutyrate dehydrogenase type 2
BDH2
Q9BV79
Trans−2−enoyl−CoA reductase, mitochondrial
MECR
Q9BZH6
WD repeat−containing protein 11
WDR11
Q9NNW7
Thioredoxin reductase 2, mitochondrial
TXNRD2
Q9UBQ7
Glyoxylate reductase/hydroxypyruvate reductase
GRHPR
Q9ULV4
Coronin−1C
CORO1C
Q04828
Aldo−keto reductase family 1 member C1
AKR1C1
Q6ZSS3
Zinc finger protein 621
ZNF621
Q8N335
Glycerol−3−phosphate dehydrogenase 1−like protein
GPD1L
Q96HP4
Oxidoreductase NAD−binding domain−containing protein 1
OXNAD1
Non−denatured protein fraction
Compound concentration(mM)
NAD.1
NADH.1
NADP.1
NADPH.1
NAD.2
NADH.2
NADP.2
NADPH.2

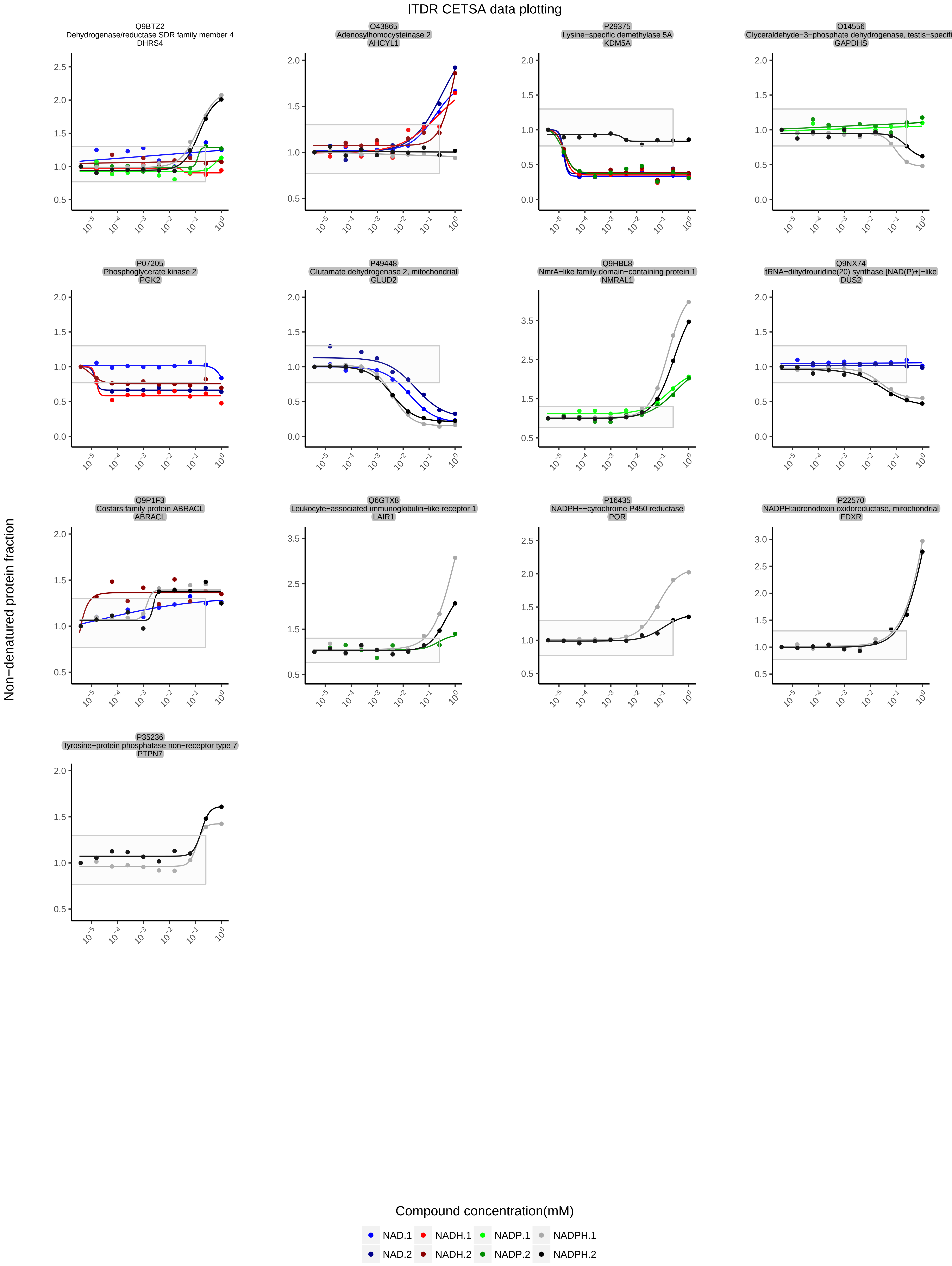
ITDR CETSA data plotting
Q9BTZ2
Dehydrogenase/reductase SDR family member 4
DHRS4
O43865
Adenosylhomocysteinase 2
AHCYL1
P29375
Lysine−specific demethylase 5A
KDM5A
O14556
Glyceraldehyde−3−phosphate dehydrogenase, testis−specifi
GAPDHS
P07205
Phosphoglycerate kinase 2
PGK2
P49448
Glutamate dehydrogenase 2, mitochondrial
GLUD2
Q9HBL8
NmrA−like family domain−containing protein 1
NMRAL1
Q9NX74
tRNA−dihydrouridine(20) synthase [NAD(P)+]−like
DUS2
Q9P1F3
Costars family protein ABRACL
ABRACL
Q6GTX8
Leukocyte−associated immunoglobulin−like receptor 1
LAIR1
P16435
NADPH−−cytochrome P450 reductase
POR
P22570
NADPH:adrenodoxin oxidoreductase, mitochondrial
FDXR
P35236
Tyrosine−protein phosphatase non−receptor type 7
PTPN7
Non−denatured protein fraction
Compound concentration(mM)
NAD.1
NADH.1
NADP.1
NADPH.1
NAD.2
NADH.2
NADP.2
NADPH.2
