# Supplementary material for: An efficient proteome-wide strategy for discovery and characterization of cellular nucleotide-protein interactions
Source: PLoS One. 2018 Dec 6;13(12):e0208273. doi: 10.1371/journal.pone.0208273 (PMC6283526; doi:10.1371/journal.pone.0208273)
Supplement: S6 Plot — We used the NADPH experiment to estimate the false negative rate in a given CETSA experiment, i.e. the number of proteins that bind to the said ligand but do not show a detectable change in thermal stability. We obtained a reviewed list of predicted human NADP binding protein based on the keyword annotation “NADP” from the protein database UniprotKB [67]. We also collected an ITDR data set at 58°C to get a better coverage of potential hits for proteins with high melting temperatures. This gave us a list of 38 proteins from the predicted NADPH binding protein list that were measured at both NADPH ITDR 52°C and 58°C datasets (S2C Fig and S8 Table), of which 11 did not come up in the list of hit proteins. One of these (ME1, P48163) gave a weak but significant response at both 52°C and 58°C and we consider this as a manually curated hit. Of the 10 remaining proteins, 8 proteins (NAXD, NAXE, PYCR3, CAT, GMPR, CRYZL1, KIAA191, NADK) had very high melting temperatures with no or < 25% melting at 58°C. These proteins would typically not be expected to respond in an ITDR at 58°C in spite of NADPH binding, but are potential responders at a higher temperature. For MICAL3 there is no direct binding data but the interaction of NADPH was reported with its close structural homolog MICAL. HSDL2 is a poorly characterised member of the short-chain dehydrogenase/reductase (SDR) subfamily. These two later proteins are candidates for non-responders in this data set. The above analysis suggests that the false negative rates for NADPH binding proteins might be very low, even below 10%. An analysis of the contribution of the 58°C experiment to hit rates showed a gain of 10 more novel hits, in addition of the 18 proteins identified in the 52°C ITDR hit list. For most of these proteins, the MDT for ITDR 52°C response was lower than at 58°C, up to 1.5 times. This is consistent with the temperature dependence of the CETSA response [25]. Interestingly, the fold changes compared to background we [file pone.0208273.s009.pdf]

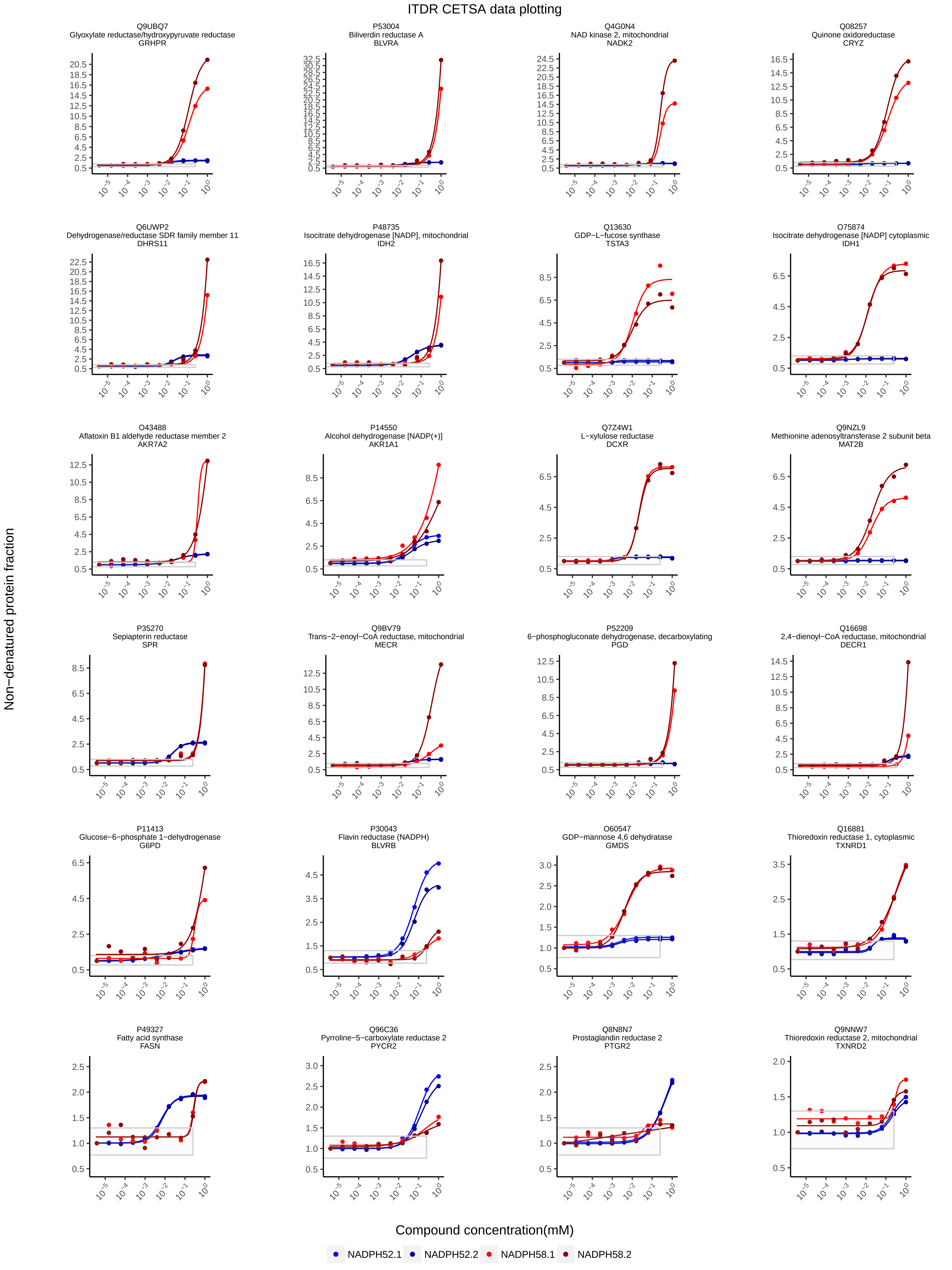
ITDR CETSA data plotting
Q9UBQ7
Glyoxylate reductase/hydroxypyruvate reductase
GRHPR
P53004
Biliverdin reductase A
BLVRA
Q4G0N4
NAD kinase 2, mitochondrial
NADK2
Q08257
Quinone oxidoreductase
CRYZ
Q6UWP2
Dehydrogenase/reductase SDR family member 11
DHRS11
P48735
Isocitrate dehydrogenase [NADP], mitochondrial
IDH2
Q13630
GDP−L−fucose synthase
TSTA3
O75874
Isocitrate dehydrogenase [NADP] cytoplasmic
IDH1
O43488
Aflatoxin B1 aldehyde reductase member 2
AKR7A2
P14550
Alcohol dehydrogenase [NADP(+)]
AKR1A1
Q7Z4W1
L−xylulose reductase
DCXR
Q9NZL9
Methionine adenosyltransferase 2 subunit beta
MAT2B
P35270
Sepiapterin reductase
SPR
Q9BV79
Trans−2−enoyl−CoA reductase, mitochondrial
MECR
P52209
6−phosphogluconate dehydrogenase, decarboxylating
PGD
Q16698
2,4−dienoyl−CoA reductase, mitochondrial
DECR1
P11413
Glucose−6−phosphate 1−dehydrogenase
G6PD
P30043
Flavin reductase (NADPH)
BLVRB
O60547
GDP−mannose 4,6 dehydratase
GMDS
Q16881
Thioredoxin reductase 1, cytoplasmic
TXNRD1
P49327
Fatty acid synthase
FASN
Q96C36
Pyrroline−5−carboxylate reductase 2
PYCR2
Q8N8N7
Prostaglandin reductase 2
PTGR2
Q9NNW7
Thioredoxin reductase 2, mitochondrial
TXNRD2
Non−denatured protein fraction
Compound concentration(mM)
NADPH52.1
NADPH52.2
NADPH58.1
NADPH58.2

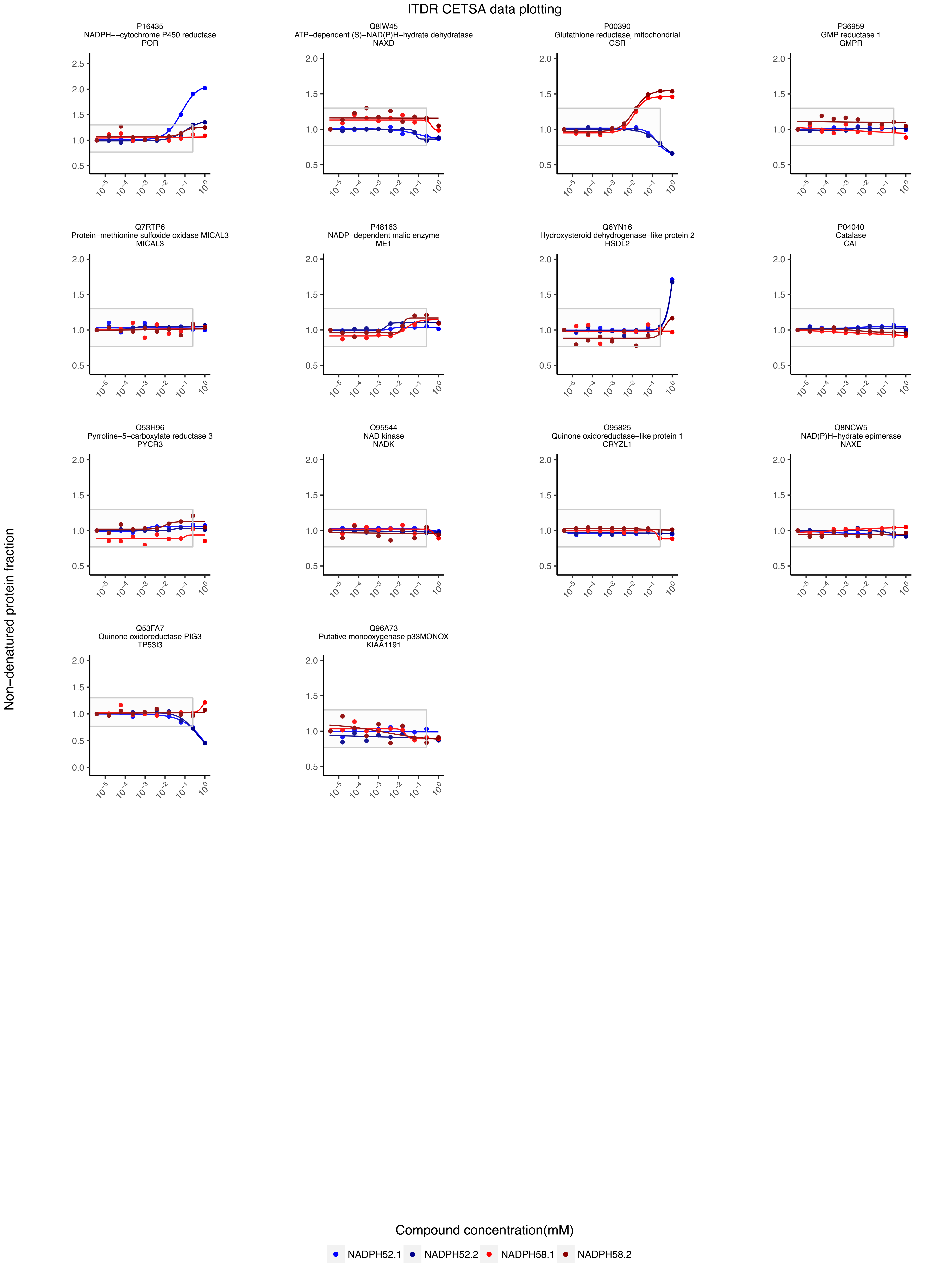

ITDR CETSA data plotting
P16435
NADPH--cytochrome P450 reductase
POR
Q8IW45
ATP-dependent (S)-NAD(P)H-hydrate dehydratase
NAXD
P00390
Glutathione reductase, mitochondrial
GSR
P36959
GMP reductase 1
GMPR
Q7RTP6
Protein-methionine sulfoxide oxidase MICAL3
MICAL3
P48163
NADP-dependent malic enzyme
ME1
Q6YN16
Hydroxysteroid dehydrogenase-like protein 2
HSDL2
P04040
Catalase
CAT
Q53H96
Pyrroline-5-carboxylate reductase 3
PYCR3
O95544
NAD kinase
NADK
O95825
Quinone oxidoreductase-like protein 1
CRYZL1
Q8NCW5
NAD(P)H-hydrate epimerase
NAXE
Q53FA7
Quinone oxidoreductase PIG3
TP53I3
Q96A73
Putative monooxygenase p33MONOX
KIAA1191
Non-denatured protein fraction
Compound concentration(mM)
NADPH52.1
NADPH52.2
NADPH58.1
NADPH58.2
