# Supplementary material for: An efficient proteome-wide strategy for discovery and characterization of cellular nucleotide-protein interactions
Source: PLoS One. 2018 Dec 6;13(12):e0208273. doi: 10.1371/journal.pone.0208273 (PMC6283526; doi:10.1371/journal.pone.0208273)
Supplement: S7 Plot — (PDF) [file pone.0208273.s010.pdf]

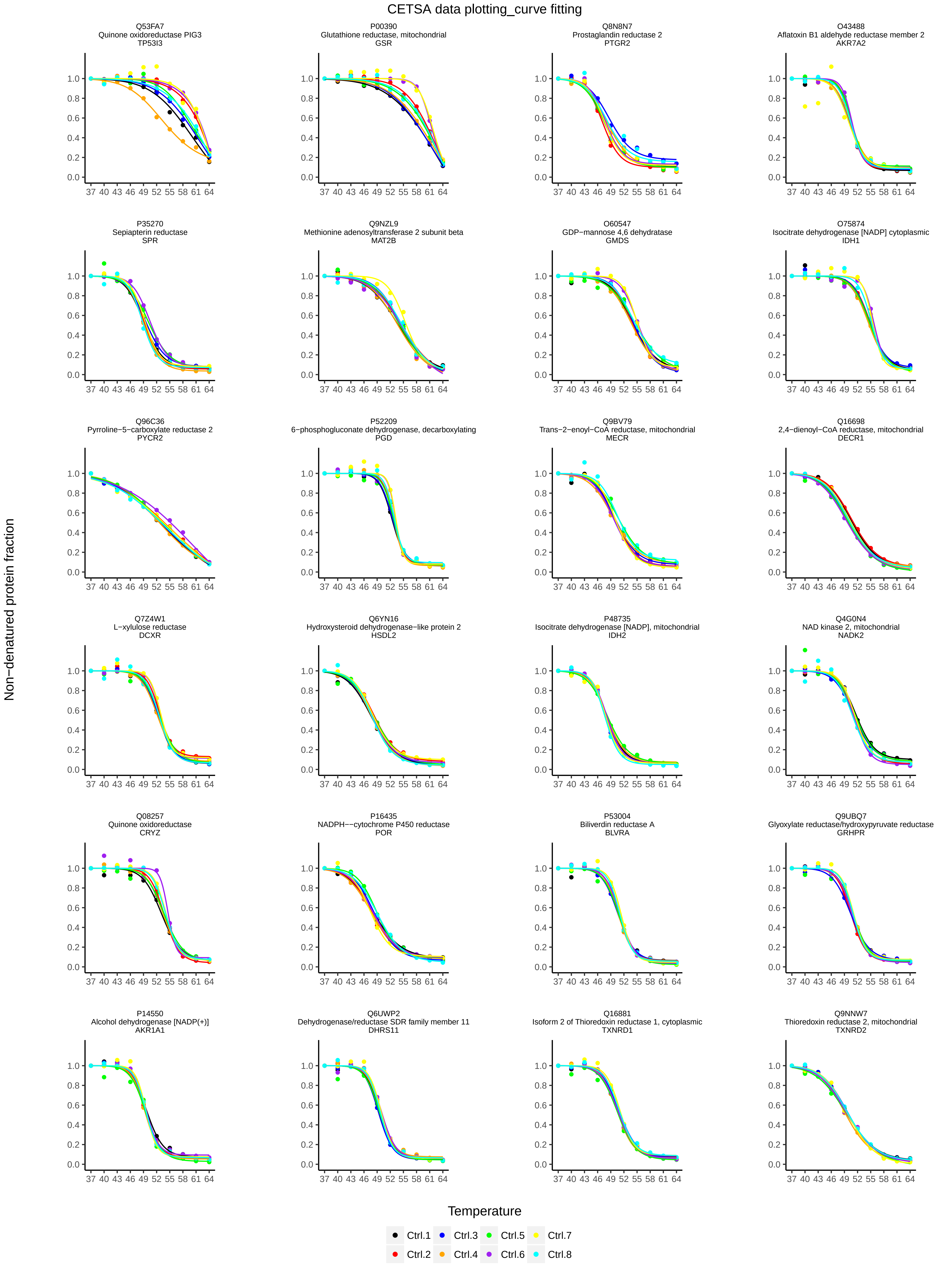
CETSA data plotting_curve fitting
Q53FA7
Quinone oxidoreductase PIG3
TP53I3
P00390
Glutathione reductase, mitochondrial
GSR
Q8N8N7
Prostaglandin reductase 2
PTGR2
O43488
Aflatoxin B1 aldehyde reductase member 2
AKR7A2
P35270
Sepiapterin reductase
SPR
Q9NZL9
Methionine adenosyltransferase 2 subunit beta
MAT2B
O60547
GDP−mannose 4,6 dehydratase
GMDS
O75874
Isocitrate dehydrogenase [NADP] cytoplasmic
IDH1
Q96C36
Pyrroline−5−carboxylate reductase 2
PYCR2
P52209
6−phosphogluconate dehydrogenase, decarboxylating
PGD
Q9BV79
Trans−2−enoyl−CoA reductase, mitochondrial
MECR
Q16698
2,4−dienoyl−CoA reductase, mitochondrial
DECR1
Q7Z4W1
L−xylulose reductase
DCXR
Q6YN16
Hydroxysteroid dehydrogenase−like protein 2
HSDL2
P48735
Isocitrate dehydrogenase [NADP], mitochondrial
IDH2
Q4G0N4
NAD kinase 2, mitochondrial
NADK2
Q08257
Quinone oxidoreductase
CRYZ
P16435
NADPH−−cytochrome P450 reductase
POR
P53004
Biliverdin reductase A
BLVRA
Q9UBQ7
Glyoxylate reductase/hydroxypyruvate reductase
GRHPR
P14550
Alcohol dehydrogenase [NADP(+)]
AKR1A1
Q6UWP2
Dehydrogenase/reductase SDR family member 11
DHRS11
Q16881
Isoform 2 of Thioredoxin reductase 1, cytoplasmic
TXNRD1
Q9NNW7
Thioredoxin reductase 2, mitochondrial
TXNRD2
0.0 0.2 0.4 0.6 0.8 1.0
37 40 43 46 49 52 55 58 61 64
Non−denatured protein fraction
Temperature
Ctrl.1
Ctrl.2
Ctrl.3
Ctrl.4
Ctrl.5
Ctrl.6
Ctrl.7
Ctrl.8

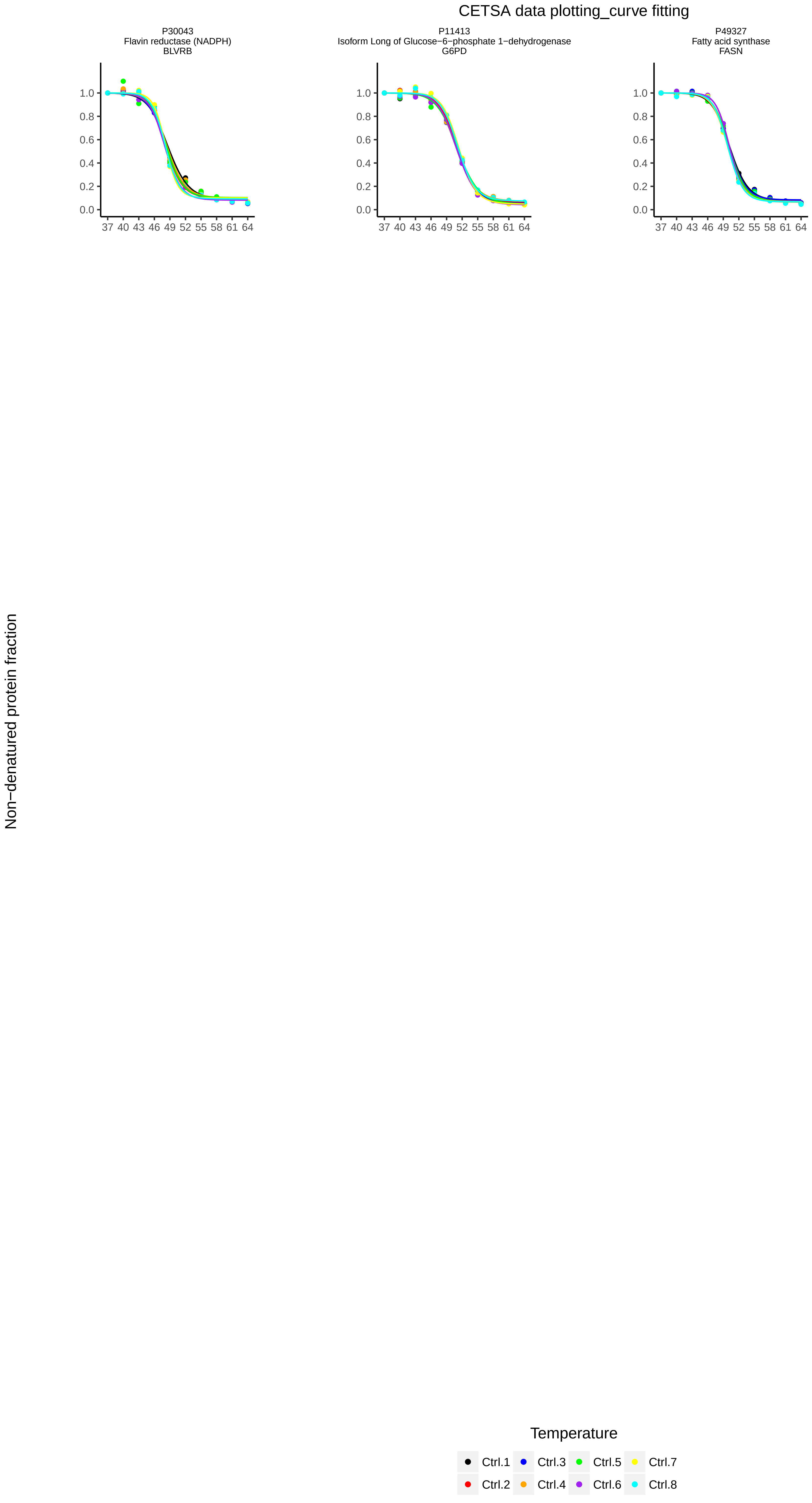
CETSA data plotting_curve fitting
P30043
Flavin reductase (NADPH)
BLVRB
P11413
Isoform Long of Glucose−6−phosphate 1−dehydrogenase
G6PD
P49327
Fatty acid synthase
FASN
Non−denatured protein fraction
Temperature
Ctrl.1
Ctrl.2
Ctrl.3
Ctrl.4
Ctrl.5
Ctrl.6
Ctrl.7
Ctrl.8

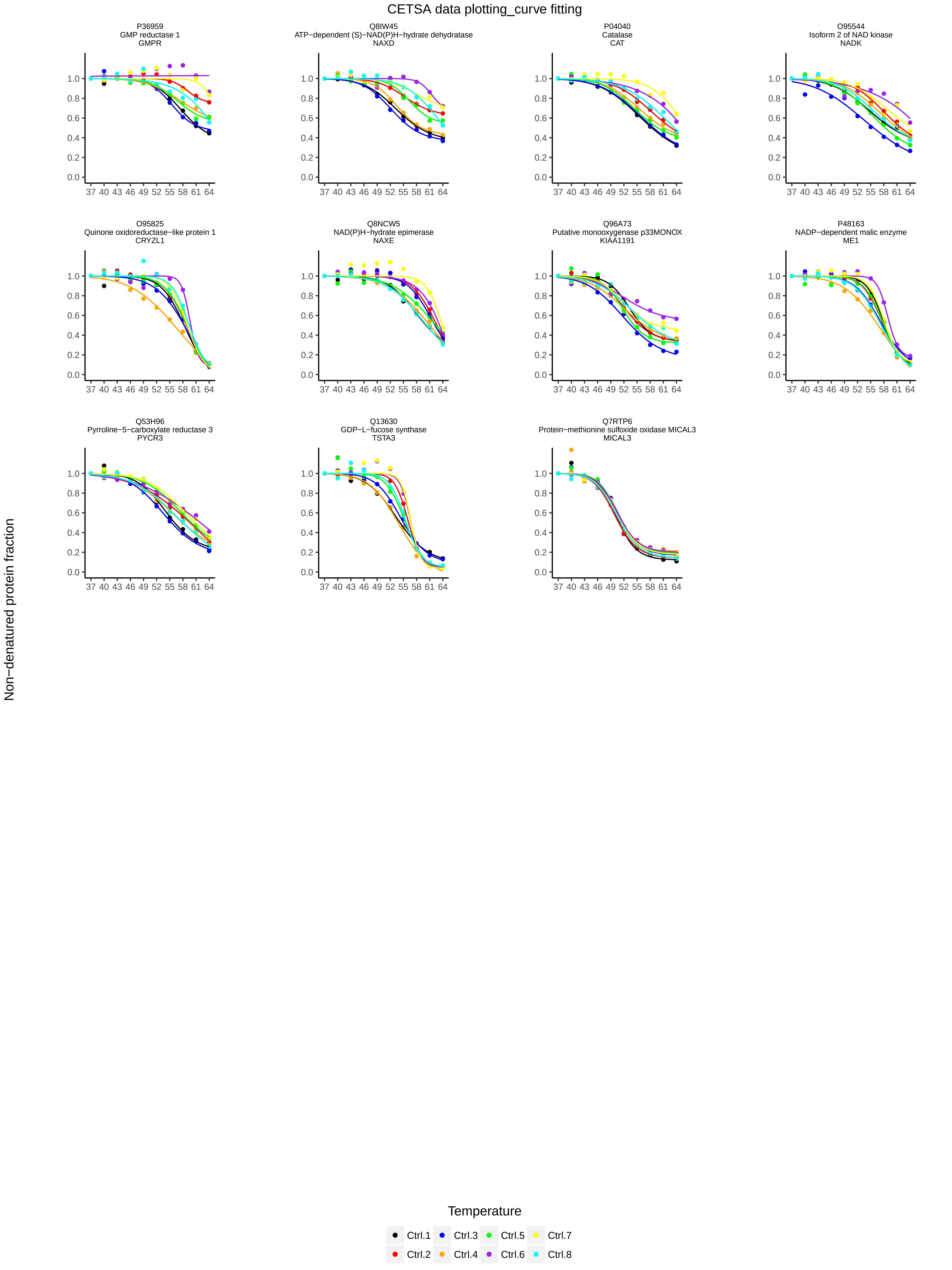
CETSA data plotting_curve fitting
P36959
GMP reductase 1
GMPR
Q8IW45
ATP−dependent (S)−NAD(P)H−hydrate dehydratase
NAXD
P04040
Catalase
CAT
O95544
Isoform 2 of NAD kinase
NADK
O95825
Quinone oxidoreductase−like protein 1
CRYZL1
Q8NCW5
NAD(P)H−hydrate epimerase
NAXE
Q96A73
Putative monooxygenase p33MONOX
KIAA1191
P48163
NADP−dependent malic enzyme
ME1
Q53H96
Pyrroline−5−carboxylate reductase 3
PYCR3
Q13630
GDP−L−fucose synthase
TSTA3
Q7RTP6
Protein−methionine sulfoxide oxidase MICAL3
MICAL3
1.0
0.8
0.6
0.4
0.2
0.0
37 40 43 46 49 52 55 58 61 64
Non−denatured protein fraction
Temperature
Ctrl.1
Ctrl.2
Ctrl.3
Ctrl.4
Ctrl.5
Ctrl.6
Ctrl.7
Ctrl.8
