# Supplementary material for: An efficient proteome-wide strategy for discovery and characterization of cellular nucleotide-protein interactions
Source: PLoS One. 2018 Dec 6;13(12):e0208273. doi: 10.1371/journal.pone.0208273 (PMC6283526; doi:10.1371/journal.pone.0208273)
Supplement: S8 Plot — Data is presented as two individual technical replicates of two biological replicates. In our preliminary experiments, we did see signs of shifts in the population of pH-sensitive proteins that were previously observed in the lysate experiments, indicating changes in intracellular pH. These pH-sensitive shifts were however not present in the subsequent more controlled experiments. (PDF) [file pone.0208273.s011.pdf]

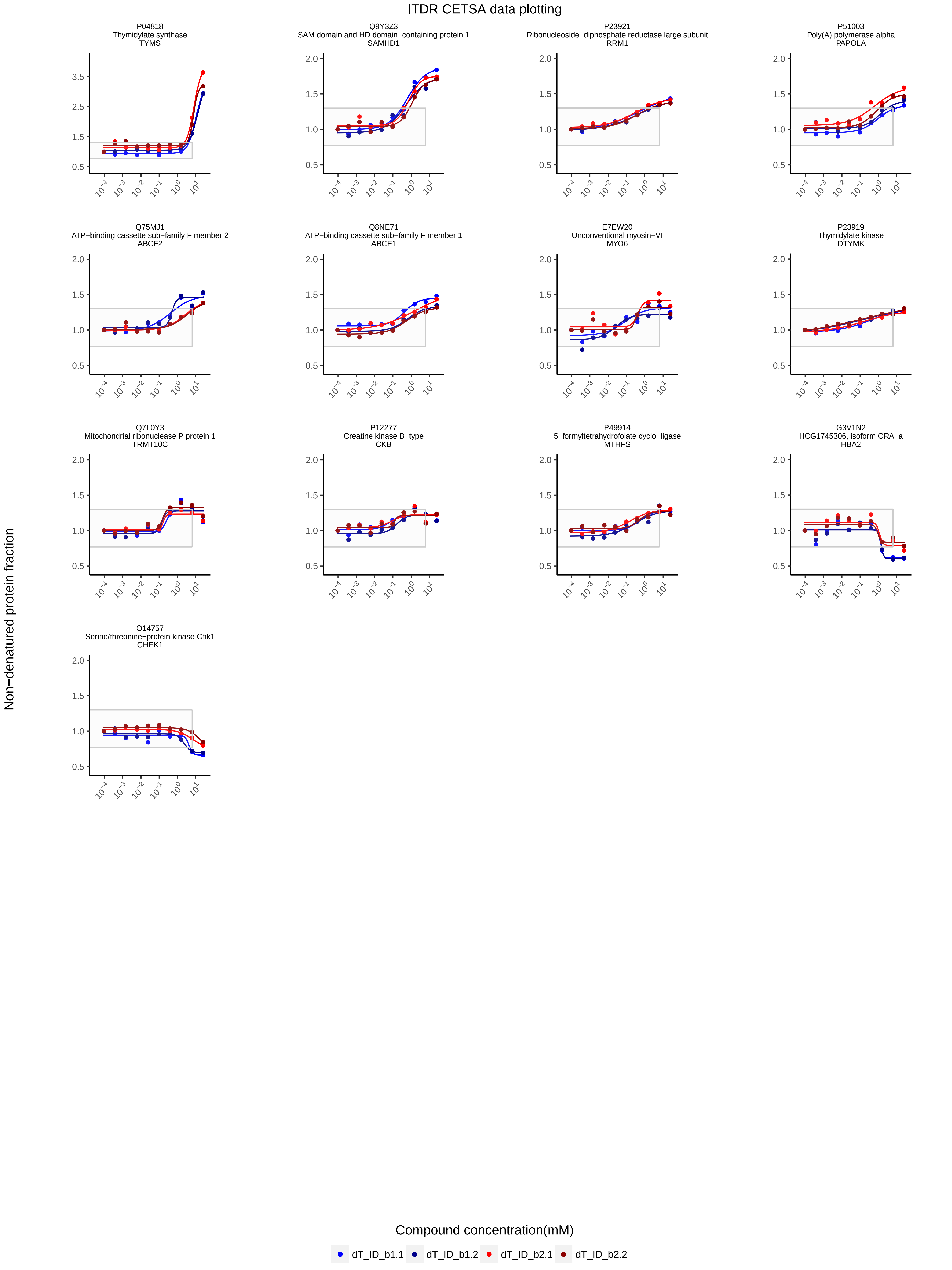
ITDR CETSA data plotting
P04818
Thymidylate synthase
TYMS
Q9Y3Z3
SAM domain and HD domain−containing protein 1
SAMHD1
P23921
Ribonucleoside−diphosphate reductase large subunit
RRM1
P51003
Poly(A) polymerase alpha
PAPOLA
Q75MJ1
ATP−binding cassette sub−family F member 2
ABCF2
Q8NE71
ATP−binding cassette sub−family F member 1
ABCF1
E7EW20
Unconventional myosin−VI
MYO6
P23919
Thymidylate kinase
DTYMK
Q7L0Y3
Mitochondrial ribonuclease P protein 1
TRMT10C
P12277
Creatine kinase B−type
CKB
P49914
5−formyltetrahydrofolate cyclo−ligase
MTHFS
G3V1N2
HCG1745306, isoform CRA_a
HBA2
O14757
Serine/threonine−protein kinase Chk1
CHEK1
Non−denatured protein fraction
Compound concentration(mM)
dT_ID_b1.1
dT_ID_b1.2
dT_ID_b2.1
dT_ID_b2.2
